# Development of molecular biomarkers for monitoring of arable crops colonization with *Methylobacterium symbioticum* SB0023/3, a methylotrophic bacterium commonly used as a biostimulant in agriculture

**DOI:** 10.3389/fpls.2026.1718185

**Published:** 2026-03-02

**Authors:** Rolf Allner, Przemyslaw Decewicz, Thomas Allner, Agata Bluszcz, Lukasz Dziewit

**Affiliations:** 1 GOBIO GmbH - Institute for Aquatic Ecology and Applied Biology, Aarbergen, Germany; 2 Laboratory of Applied Microbial Ecology, Institute of Bioengineering, Faculty of Biology, University of Warsaw, Warsaw, Poland

**Keywords:** biomonitoring, biostimulant, *Methylobacterium symbioticum*, plant colonization, real-time PCR

## Abstract

**Introduction:**

The intensive use of synthetic fertilizers has led to increased nitrous oxide emissions, declining soil fertility, and reduced biodiversity. Biological alternatives, such as the use of endophytic bacteria to improve plant growth, are promising alternatives but require reliable monitoring tools to assess colonization success and biological effectiveness under field conditions. One of the most commonly used microbial biostimulants is *Methylobacterium symbioticum* SB0023/3; thus, monitoring the efficacy of inoculation and maintenance of this strain is required for adequate evaluation of fertilization practices.

**Methodology:**

The resequencing of the genome of *M. symbioticum* SB0023/3, followed by comparative genomics and functional annotation were performed. Specific real-time PCR primers were developed and validated for strain-specific detection. The colonization of various crops (wheat, corn, rapeseed, peas, and tomatoes) was tested under controlled conditions using developed molecular markers.

**Results:**

The resequencing of the SB0023/3 genome revealed novel genetic content and updated previous records. The resequenced genome showed 121 novel regions with 165 protein-coding genes and five tRNA. Based on the newly obtained genome, two highly specific biomarker genes (*copG* and *ubik*) were identified and, together with the (*Methylobacterium* spp./methylotrophs-specific) *xoxF* gene, validated for their strain/genus-specificity. The developed real-time PCR assays using *copG* and *ubik* biomarkers demonstrated high specificity for *M. symbioticum* SB0023/3, distinguishing it from related species. In contrast, the *xoxF* gene showed relaxed specificity and cannot be used for SB0023/3 detection. Successful endophytic colonization was confirmed in all tested crops, with high detection rates exceeding 80% in tomatoes. Classical culturing on a novel nitrogen-free medium additionally confirmed colonization, with the same validating the real-time PCR assays.

**Discussion:**

This study provides a robust, genome-informed molecular detection system for monitoring *M. symbioticum* SB0023/3 in crops. The presented approach enables direct detection from plant tissues, facilitating studies on colonization dynamics and biosafety. This methodology can be extended to other microbial biostimulants, supporting sustainable agricultural practices.

## 1 Introduction

The intensive use of synthetic fertilizers has been a major driver of the drastic increase in nitrous oxide emissions (Tian et al., 2020). It is also recognized as an important contributor to the decline in soil fertility (Tripathi et al., 2020) and the alteration or loss of natural biodiversity (Pahalvi et al., 2021), one of the most pressing global crises (Newbold et al., 2016).

The substitution of synthetic nitrogen fertilizers with biological nitrogen sources, therefore, represents both a promising strategy and a major challenge on the path toward sustainable agriculture (Mus et al., 2016). Among the various approaches, the inoculation of non-leguminous crops with endophytic diazotrophic bacteria has been extensively investigated as a means to improve plant nitrogen supply (Soumare et al., 2020). Field trials are critical to evaluate both colonization and functional efficacy of these bacteria under agricultural conditions. While numerous greenhouse and laboratory studies demonstrate plant growth–promoting effects of microbial biostimulants with respect to yield and quality (Silveira et al., 2016), comparatively only a few field trials have provided direct evidence of successful endophytic colonization. Moreover, field results are often inconsistent, with instances showing limited or no measurable effects. In some cases, improvements in plant performance are used as indirect evidence of colonization, but direct confirmation is frequently lacking (De Almeida Lopes et al., 2016; Ahmad et al., 2022; Torres Vera et al., 2024; Tsipinana et al., 2024). The scarcity of rigorously documented field studies is largely due to the absence of simple, reliable, and strain-specific detection methods.

Traditional approaches, such as enumeration of microorganisms on selective nutrient media, generally fail to differentiate natural from artificially introduced endophytic colonization (Zinniel et al., 2002). While 16S rRNA gene sequencing provides robust genus-level identification, species-level resolution is often constrained by the quality of available reference databases and methodological biases, including interference from plant DNA, intragenomic variation in 16S rRNA operons, and PCR artifacts (Church et al., 2020). This highlights the urgent need to develop new, strain-specific molecular biomarkers and detection systems suitable for monitoring inoculated microorganisms with biofertilizing and/or biostimulating potential in agricultural settings.

One of the most widely used microbial biofertilizers/biostimulants, with applications covering more than 2 million hectares worldwide in 2024 (M. Dzikowski, Corteva Agriscience, personal communication, July 8th, 2025), contains *Methylobacterium symbioticum* SB0023/3, a Gram-negative alphaproteobacterium. This species is characterized by its pink pigmentation, attributable to carotenoid biosynthesis, and its ability to utilize C1 carbon sources such as methanol (Toyama et al., 1998; Van Dien et al., 2003). *M. symbioticum* can grow in nitrogen-free media, although its nitrogen fixation mechanism remains unclear (Giller et al., 2025). Nonetheless, its plant growth-promoting effects are well documented, supporting its continued use in crop production (Agafonova et al., 2013; Dourado et al., 2015; Pascual et al., 2020; Juma et al., 2022; Pappalettere et al., 2024). However, its monitoring in the field and assessment of colonization efficiency remain challenging.

In this study, we performed resequencing of *M. symbioticum* SB0023/3 (first reported genomic sequence: GenBank acc. no. GCF_902141845.1), intending to refine the genomic reference and identify additional genetic features relevant to its ecological function. Particular emphasis was placed on the systematic identification of molecular biomarkers that could facilitate strain-specific biomonitoring, which fills the significant knowledge gap for the application of that agriculturally-relevant strain. These biomarkers are designed to be compatible with high-throughput laboratory methods and adaptable to diverse agricultural contexts, including various crop species, biological matrices, and potential vectors that may influence the bacterium’s distribution in agroecosystems. Establishing such a methodological framework is critical for the development of robust molecular tools to enable precise tracking of *M. symbioticum* SB0023/3 during field applications and to advance our understanding of its ecological interactions. It is also important to emphasize that the development of strain-specific biomarkers is critical, as it provides an advantage over biomarkers specific to an entire functional group or genus (e.g., the *xoxF* gene), as well as over general bacterial biomarkers (e.g., the 16S rRNA gene). The latter requires further bioinformatic analysis and lacks specificity toward the target group of interest, instead providing insight into the overall environmental biodiversity. Additionally, the use of strain-specific biomarkers and molecular methods offers advantages over classical approaches, including cultivation-based methods, which are time-consuming and costly. Overall, the study aims to fill a significant methodological gap by providing a genomic, strain-specific tool for directly tracking *M. symbioticum* SB0023/3 in plants, which, to our knowledge, has been largely lacking in previous work on *Methylobacterium* and other bacterial biostimulants. Furthermore, this work proposes a pipeline for the general implementation of the presented methods for use in biomonitoring.

## 2 Materials and methods

### 2.1 Bacterial strains and cultivation conditions

*M. symbioticum* SB0023/3 was obtained from Corteva Agriscience GmbH, Munich, Germany. Lyophilized *M. symbioticum* (1.7 mg/ml) was dissolved in DPBS buffer (Carl Roth, Karlsruhe, Germany). Then the strain was cultivated on a nitrogen-free medium (Cf-Nf). Pink colonies were seen after 8 days of incubation at 28 °C. The taxonomy of the strain was confirmed via 16S rDNA sequencing.

In this study, a novel nitrogen-free medium – Cf-Nf – was developed, and methanol was used as a single carbon source in this medium. The Cf-Nf medium was composed of: 17 g/L Noble Agar (Thermo Scientific, Darmstadt, Germany), K_2_HPO_4_ (0.5 g/L) (Merck, Darmstadt, Germany), MgSO_4_ × 7H_2_O (0.2 g/L) (Carl Roth, Karlsruhe, Germany), FeSO_4_ × 7H_2_O (0.1 g/L) (Carl Roth), CaCl_2_ × 2H_2_O (0.01 g/L) (Carl Roth), NaCl (0.1 g/L) (Life Technologies, Darmstadt, Germany). To this, 2 mL micronutrient solution containing CuSO_4_ × 5H_2_O (0.4 g/L) (Carl Roth), ZnSO_4_ × 7 H_2_O (0.12 g/L) (Carl Roth), MgSO_4_ × 7 H_2_O (0.2 g/L) (Carl Roth), H_3_BO_3_ (1.4 g/L) (Merck), Na_2_MoO_4_ × 2H_2_O (1 g/L) (Carl Roth), MnSO_4_ × H_2_O (1.5 g/L) (Carl Roth); 2 mL bromothymol blue solution (0,5g/100 mL 0,2M KOH) (Carl Roth); 4 mL Fe(III)-EDTA (1.64% final concentration) (Carl Roth); and 1 mL vitamin solution (10 mg biotin (Carl Roth) and 20 mg pyridoxal HCl (Carl Roth) in 100 mL H_2_O) was added. The micronutrient and vitamin solutions were autoclaved individually prior to their incorporation into the base medium. The pH level of the Cf-Nf medium was adjusted to 7. The Cf-Nf medium was autoclaved at 121 °C for 20 minutes. It was cooled down to 55 °C, and 2% methanol was added before plating.

The reference strains of *M. radiotolerans* DSM 760, *M. mesophilicum* DSM 1708 and *M. dankookense* SW08-7 (DSM 22415) were obtained from DSMZ (German Collection of Microorganisms and Cell Cultures) and cultured on nutrient agar [peptone from casein (10 g/L) (Carl Roth), yeast extract (5 g/L) (Carl Roth) and agar (15 g/L) (Carl Roth)]. For the DSM 760 and DSM 1708 strains, no growth was seen on the Cf-Nf selective medium. *M. dankookense* showed comparable growth to *M. symbioticum* on the selective nutrient medium, but was also cultivated on full nutrient medium, as were the other reference strains.

### 2.2 DNA isolation

*M. symbioticum* SB0023/3 was cultivated in R2A medium at 20 °C with shaking at 160 rpm for 48 hours. After incubation, the culture was centrifuged at 6,000 × g for 5 min (benchtop centrifuge 5424R, Eppendorf, Hamburg, Germany), and the resulting cell pellet was used for genomic DNA extraction with the Bacterial & Yeast Genomic DNA Purification Kit (EURx, Gdansk, Poland) according to the manufacturer’s instructions. To enhance cell lysis efficiency, lysozyme (Merck, Darmstadt, Germany) was added during the lysis step to a final concentration of 1 mg/mL. DNA concentration was measured using a Qubit™ 2.0 Fluorometer (Invitrogen, Carlsbad, CA, USA), while purity was assessed with a NanoReady Touch Microvolume Spectrophotometer (Life Real, Hangzhou, China) and further confirmed by electrophoresis in a 0.8% agarose gel.

### 2.3 Genomic DNA sequencing and processing

Whole-genome resequencing of *M. symbioticum* SB0023/3 was performed using both short-read (Illumina) and long-read (Oxford Nanopore Technologies, ONT) sequencing approaches. Illumina sequencing was carried out by Eurofins Genomics GmbH (Ebersberg, Germany). Libraries were prepared following the standard Eurofins protocol and sequenced on an Illumina NovaSeq 6000 platform with an S4 flow cell, generating 2 × 150 bp paired-end reads. For ONT sequencing, libraries were prepared using the Native Barcoding Kit 24 v14 (Oxford Nanopore Technologies) according to the manufacturer’s protocol, and sequencing was performed on an R10.4 flow cell with a MinION Mk1B device.

The Illumina dataset comprised 13.67 million reads with a total length of 2 Gbp. Raw reads were processed with fastp v0.23.4 in paired-end mode using the parameters --cut_tail --detect_adapter_for_pe --trim_poly_x --cut_window_size 10 --cut_mean_quality 25. The ONT dataset comprised 245 thousand reads, including 7300 duplex ones, with a total length of 576 Mbp. The reads were basecalled with DORADO v0.9.0 using dna_r10.4.1_e8.2_400bps_sup@v5.0.0 model, followed by barcode trimming and reads filtering based on the length to keep only reads longer than 2000 bp. Filtered reads were assembled with Unicycler v0.5.1 wrapping SPAdes v4.0.0 (Prjibelski et al., 2020), Racon v1.5.0 (Vaser et al., 2017), minimap2 v2.28 (Li, 2021), and miniasm v0.3 [https://github.com/lh3/miniasm]. The coverage was calculated using CoverM v0.7.0 (Aroney et al., 2025), wrapping samtools 1.19 (Danecek et al., 2021) and with either bwa-mem2 v2.1.1 (Vasimuddin et al., 2019) or minimap2 v2.28 for short and long reads, respectively. Complete plasmid replicons were reoriented using the dnaapler v1.3 (Bouras et al., 2024).

### 2.4 Genome annotation

The cleaned genome assembly was automatically annotated using bakta v1.7.0 (database v5.1) (Schwengers et al., 2021) and eggnog-mapper v2.1.12 (Huerta-Cepas et al., 2019; Cantalapiedra et al., 2021). The results were subsequently manually evaluated and refined with MAISEN v2.1.3 (Dziurzynski et al., 2021). During annotation, the following tools and databases were employed: tRNAscan-SE v2.0 (Chan et al., 2021), Aragorn (Laslett and Canback, 2004), Infernal (Nawrocki and Eddy, 2013), PilerCR (Edgar, 2007), Pyrodigal (Hyatt et al., 2010; Larralde, 2022), Diamond (Buchfink et al., 2021), BLAST+ (Camacho et al., 2009), PyHMMER (Eddy, 2011; Larralde, 2022), AMRFinderPlus (Feldgarden et al., 2021), HHPred (Hildebrand et al., 2009; Zimmermann et al., 2018), MMseqs2 (Steinegger and Söding, 2017), UniProt (The UniProt Consortium et al., 2021), RCSB Protein Data Bank (Burley et al., 2021), ECOD (Cheng et al., 2014), SCOPe (Chandonia et al., 2022), TIGRFAMs (Li et al., 2021), and the Conserved Domain Database (Wang et al., 2023).

### 2.5 Comparative genomics

Comparative genomics between the genomic sequences of *M. symbioticum* SB0023/3, i.e., GenBank acc. no. GCF_902141845.1, and the newly resequenced genome was performed using Panaroo v1.5.0 (Tonkin-Hill et al., 2020) with the parameters --clean-mode sensitive --threshold 0.98 --family_threshold 0.7, nucleotide BLAST using e-value 1e-20 as the threshold, and MMseqs2 clustering with 80% sequence coverage and identity thresholds. Additional searches were conducted against RefSeq-annotated genomes (Li et al., 2021) and the NCBI NR database using the parameters -s 7.5 -c 0.3 -e 1e-3 --cov_mode 0 (Sayers et al., 2022). The RefSeq-annotated assembly was downloaded from the RefSeq database and used as a reference for comparative analysis. Whole-genome comparisons at the nucleotide level were carried out to identify regions that were not conserved between the two assemblies.

### 2.6 Real-time PCR and validation of the specificity of the molecular markers

PCR primers were synthesized by Eurofins Genomics (Cologne, Germany). Amplifications were performed in 20 µl reaction volumes using a StepOnePlus Real-Time PCR System (Applied Biosystems, Thermo Fisher Scientific, Bensheim, Germany). Each reaction contained 100 ng of genomic DNA, 10 µl of PowerUp SYBR Green Master Mix (Thermo Fisher Scientific Baltics UAB), and 0.4 nM of each primer. Three primer pairs were used: (i) GoBio_Xoxf_f (5′-GGTCGAGCTTGGGATCGTAG-3′)/GoBio_Xoxf_r (5′-GAAGAAGGGCGAGACCAACA-3′); (ii) GoBio_CopG_f (5′-TCATCACCCAAGCCAACCAG-3′)/GoBio_CopG_r (5′-TCATGGTCGATCCGTCCTCT-3′); (iii) GoBio_Ubik_f (5′-GTTCATCGCCCTTGAGGTAG-3′)/GoBio_Ubik_r (5′-GTTCCTTGCGAATGCGGG-3′).

The thermal cycling protocol consisted of an initial denaturation at 95 °C for 2 min, followed by 40 cycles of 95 °C for 30 s, 60 °C for 30 s, and 72 °C for 45 s. Melt curve analysis was subsequently performed by increasing the temperature from 72 °C to 95 °C at a rate of 0.3 °C/s, with continuous fluorescence acquisition.

Melt curve analysis was used to confirm the presence of *M. symbioticum* in the tested samples. During amplification, SYBR Green binds to double-stranded DNA and emits fluorescence. Gradual denaturation of the amplicons released SYBR Green, resulting in decreased fluorescence. Plotting fluorescence intensity against temperature produced a melt curve characteristic of each amplicon. The melting profile was influenced by amplicon length, sequence composition, GC content, and strand complementarity. Samples were considered positive for *M. symbioticum* when the cycle threshold (Ct) value was ≤30 and the melting temperature (Tm) differed by no more than 0.5 °C from that of the positive control.

To evaluate the strain specificity of the molecular markers and the detection protocol, three additional *Methylobacterium* species: *M. radiotolerans* DSM 760, *M. mesophilicum* DSM 1708, and *M. dankookense* SW08–7 were used.

### 2.7 Plant cultivation and leaf samples collection

Experiments were carried out in a climate chamber (Model FPG-450, Faithful Instrument [Hebei] Co., Ltd., Cangzhou, China). Five arable plant species were selected for experimentation: wheat (*Triticum aestivum*), corn (*Zea mays*), oilseed rape (*Brassica napus*), pea (*Pisum sativum*), and tomato (*Solanum lycopersicum*). Seeds were cultivated in a hydroponic medium inside the chamber for 45 days under a 16 h light/8 h dark photoperiod and 60% relative humidity. Plants were divided into two groups: a control group and a treatment group inoculated with *M. symbioticum* SB0023/3. The inoculum was prepared by suspending *M. symbioticum* SB0023/3 in DPBS (Dulbecco’s Phosphate-Buffered Saline; VWR International, Belgium/France) at a concentration of 10,000 CFU/ml. The suspension was applied to the leaves of test plants using a standard hand pump sprayer.

### 2.8 Leaf samples preparation and DNA isolation

Leaves from untreated and inoculated plants were collected 14 (n = 6) and 28 (n = 3) days after treatment. Each sample originated from a different plant but from the same developmental level (youngest fully developed leaves). Due to the rapid growth of maize and oilseed rape, the available space in the climate chamber became limited; relocating plants would have compromised comparability, and therefore data from the second time point are not shown for those crops. No plant was sampled more than once to avoid potential effects of leaf removal. To prevent cross-contamination, untreated control plants were cultivated in a second climate chamber of the same design and with identical environmental conditions.

**Figure 1:**
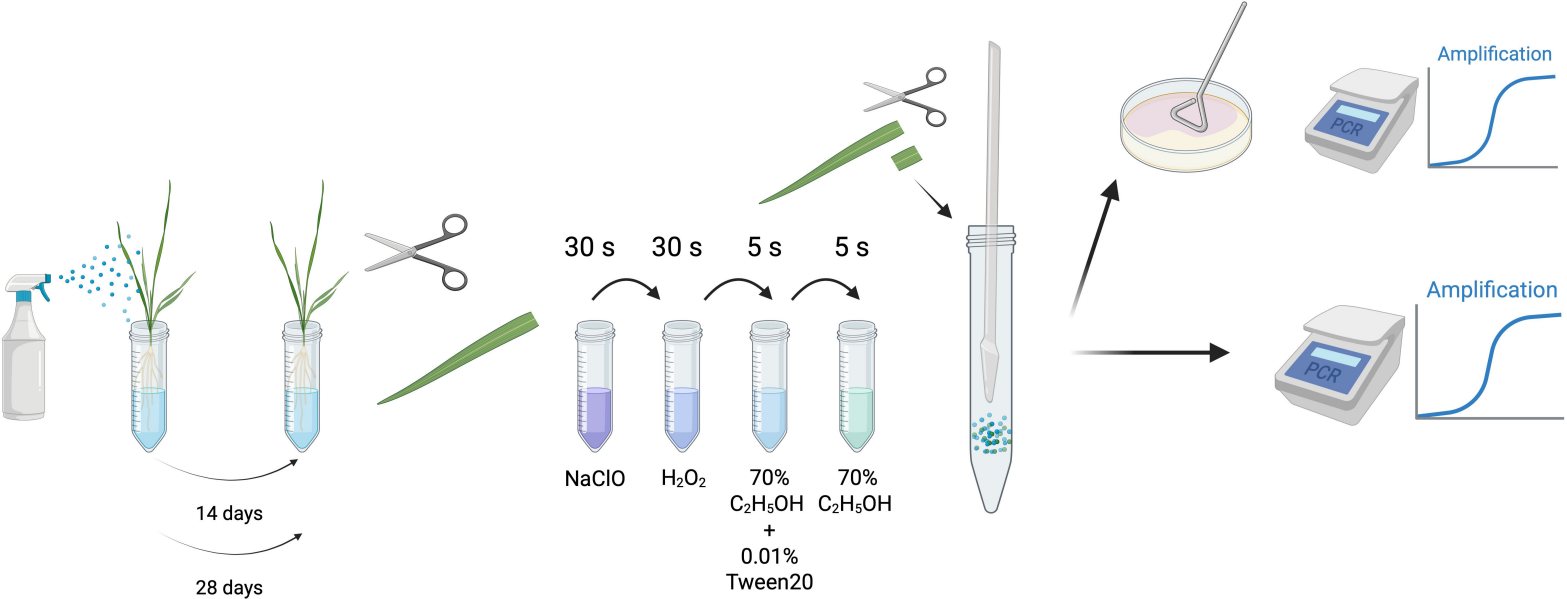
Schematic representation of sampling and disinfection of inoculated leaf samples. Created in BioRender. Allner, R. (2025) https://BioRender.com/oijk4u1.

Leaf samples underwent surface sterilization through sequential washes: 3% sodium hypochlorite (Life Technologies) for 30 s, 3% hydrogen peroxide (35%, Carl Roth) for 30 s, 70% ethanol (VWR, Darmstadt, Germany) with 0.01% Tween-20 (Carl Roth) for 5 s, and 70% ethanol (VWR) for 5 s (**Figure 1**). Samples were dried with sterile filter paper, transferred into 15 ml Falcon tubes (Roth, Karlsruhe, Germany), and kept on dry ice. Homogenization was performed with a sterile pestle, after which 2 ml of DPBS buffer was added. Samples were incubated at 28 °C for 12 h. On the following day, leaf extracts were plated onto CF-Nf agar. The remaining extract was filtered through sterile gauze into a new 15 ml Falcon tube, supplemented with 2 ml DPBS containing 2% methanol, and incubated for another 12 h at 28 °C.

On the third day, samples were vortexed for 20 s and centrifuged (Heraeus Multifuge 4 KR, Thermo Scientific) at 4,650 × g for 25 min. The supernatant was discarded, and the pellet was resuspended in 100 µl nuclease-free distilled water. For DNA extraction, 12 µl of 20% Chelex 100 Resin (Sigma-Aldrich, St. Louis, MO, USA) was mixed with 38 µl of the leaf extract in 500 µl PCR tubes. Tubes were incubated at 95 °C for 15 min, cooled at 6 °C for 3 min, vortexed briefly, and centrifuged at 16,000 × g for 2 min. The supernatant was transferred to 200 µl PCR tubes, and the extracted DNA was stored at -20 °C until further analyses.

## 3 Results

### 3.1 Characterization of the resequenced *M. symbioticum* SB0023/3 genome

The hybrid assembly of the *M. symbioticum* SB0023/3 resulted in the reconstruction of a complete genome of a total length of 6,203,152 bp (**Table 1**). The genome is composed of a single circular chromosome, two circular plasmids (pMSB0023-3_1 and pMSB0023-3_2), and a prophage (pMSB0023-3_3) existing in the genome as an integrated prophage or an episome. The GC content varies from 65.9 – 70.3% with an average of 70.2%. Total coding density was estimated as 85.8%. The resequenced genome comprised 5,744 protein-coding genes, 61 tRNAs, 15 rRNAs, 12 ncRNAs, and 1 tmRNA (**Table 1**). Among the protein-coding genes, 533 were annotated as hypothetical proteins, and 289 carried domains of unknown function.

**Table 1:** General characteristics of the resequenced *M. symbioticum* SB0023/3 genome.

Genomic feature	Complete genome	Chromosome	pMSB0023-3_1	pMSB0023-3_2	Prophage as episome (genome location: 5,964,342.6,021,680)
Length (bp)	6,203,152	6,021,680	123,595	57,877	57,339
Short/Long reads depth	316×/83×	313×/84×	539×/170×	232×/93×	492×/158×
GC-content (%)	70.2	70.2	66.6	65.9	69.7
CDSs	5,744	5,564	127	54	84
tRNAs	61	60	0	1	1
tmRNAs	1	1	0	0	0
rRNAs	15	15	0	0	0
nsRNAs	12	12	0	0	0
Regulatory ncRNAs	19	19	0	0	0

While complete genomes of all replicons were successfully reconstructed, it must be noted that the prophage genome (chromosome coordinates: 5,964,342.6,021,680) was also observed in an episome state. The assembly performed by Unicycler and further evaluation of the assembly graph indicated a dual-state of the prophage genome in part of the cell population, i.e., integrated within a chromosome or excised and circularized as an episome. This observation was confirmed by manual evaluation of reads overlapping the ends of the prophage genome as an episome, the adjacent regions of the chromosome of the prophage integration site, as well as reads stretching over the SB0023/3 chromosome and prophage genomes when integrated. Additionally, the coverage of the prophage region was approx. 1.6× higher compared to the chromosome.

Resequencing of the SB0023/3 genome identified 121 regions (at least 10-bp-long) across the chromosome that exclusively lacked significant similarity (e-value > 1e−10) to the previously deposited genome of *M. symbioticum* SB0023/3 (GenBank acc. no. GCF_902141845.1), with a total length of 88,574 bp. These novel regions contained predicted 165 protein- and 5 tRNA-coding genes (**Supplementary Material Table 1**). Comparative analysis with Panaroo revealed functional annotation discrepancies between the two assemblies, including the presence or absence of certain proteins. In total, 7 proteins were unique to the original SB0023/3 assembly, whereas 144 proteins were unique to the resequenced genome (**Supplementary Material Table 2**). The observed discrepancies in functional annotation strongly suggest discontinuity of the original only-Illumina-based sequencing due to repetitive regions and the presence of mobile genetic elements and related proteins. Among the overrepresented proteins in the complete genome, there are 16 insertion sequences (IS*110* – 8, IS*256* – 4, IS*3* – 2, IS*3*/IS*911* – 2), 5 integrase domain-containing proteins, and plasmid replication protein RepA. Importantly, proteins involved in the cell surface and envelope composition, in particular extracellular polysaccharides (EPS) metabolism, influencing the biofilm formation and the host-plant interaction of the SB0023/3 strain were also identified. These include polysaccharide biosynthesis/export proteins (Tripathi et al., 2020) together with polysaccharide chain length determinant domain-containing protein (Tian et al., 2020), UDP-glucose 6-dehydrogenase (Pahalvi et al., 2021), porin (Tripathi et al., 2020), transglycosylase (Tripathi et al., 2020), FtsI peptidoglycan transpeptidase (Tian et al., 2020), and DegP and DegP-like periplasmic peptidase (Tripathi et al., 2020). The functions of other proteins, among others, relate to motility (e.g., flagellar hook-length control FliK – 2), chemotaxis (CheW – 2), stress response (e.g., SOS-response peptidases – 2), DNA repair (e.g., DNA Pol Y or RecF proteins), and virulence/secretion (e.g., TssA of T6SS). On the other hand, the complete assembly is missing an ANT(3”)-Ia aminoglycoside nucleotidyltransferase (WP_001206316.1). Although the protein sequence does not present any significant similarity (e-value 0.01) to reference sequences of the Comprehensive Antibiotic Resistance Database (McArthur et al., 2013), there is a wide range of nearly identical proteins in the NCBI nr database.

**Figure 2:**
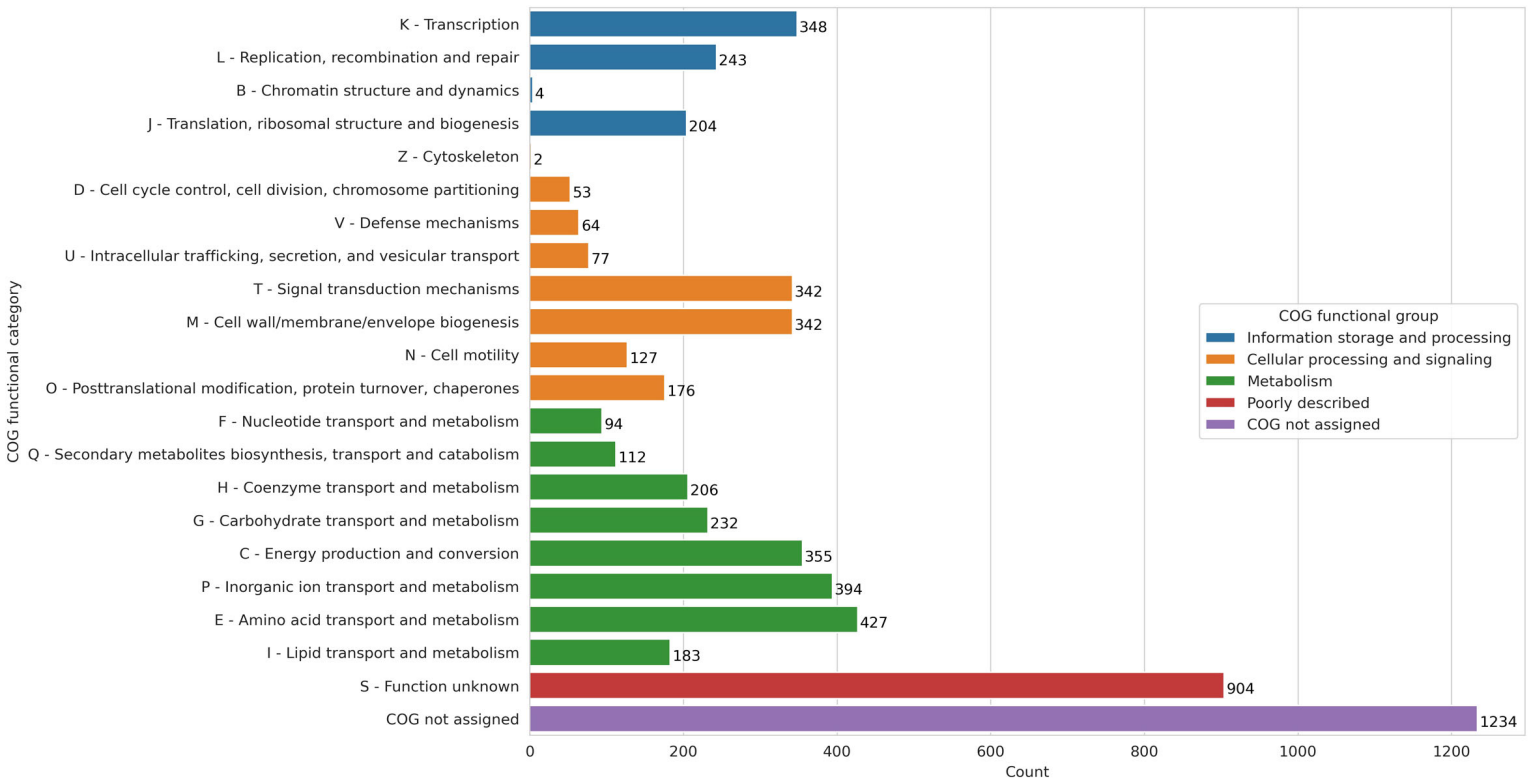
COG categories assigned to genes found within the resequenced genome of *M. symbioticum* SB0023/3 and their respective counts.

In the resequenced genome, genes were assigned to clusters of orthologous groups (COGs) (**Figure 2**). Comparison with the COG database revealed that approximately 1,999 genes (30%) were associated with metabolism-related categories. 1,246 genes (21%) lacked functional assignment, and 890 genes (15%) were annotated as encoding proteins of unknown function. In addition, 1,185 genes (20%) were assigned to cellular processes and signaling, while 791 genes (13%) were associated with information storage and processing. Within the metabolic category, subgroup E (amino acid transport and metabolism) was the most abundant, comprising 430 genes, surpassed only by the group of genes with uncharacterized functions. Subgroups P (inorganic ion transport and metabolism) and C (energy production and conversion) were the next most represented, with 398 and 352 genes, respectively, underscoring their importance in the metabolic capacity of *M. symbioticum* (**Figure 2**).

### 3.2 Development of *xoxF*-specific PCR primers

The *xoxF* gene encodes a methanol dehydrogenase, which plays a central role in methanol utilization and can thus serve as a marker for the presence of methylotrophic bacteria, particularly members of the genus *Methylobacterium*, and not specifically *M. symbioticum* SB0023/3 (Alessa et al., 2021). We hypothesized that combining *xoxF*-based molecular detection with, e.g., a cultivation approach employing a selective medium with methanol as the sole carbon source would enable reliable biomonitoring of methylotrophic and diazotrophic bacteria in plants and soil. The *xoxF* gene is highly conserved in *Methylobacterium*, and therefore the *xoxF1* gene (*locus_tag* MET9862_RS14105; NCBI acc. no. NZ_CABFPH010000037), encoding the lanthanide-dependent methanol dehydrogenase XoxF1 of *M. symbioticum* SB0023/3, was selected as the target for primer design. The primer design criteria were as follows: (i) PCR amplicon length > 300 bp, suitable for real-time PCR analyses; (ii) primer self-complementarity between 0 and 4; (iii) GC content between 40% and 60%. As a result, the following primer pair was designed: GoBio_Xoxf_f (5′-GGTCGAGCTTGGGATCGTAG-3′)/GoBio_Xoxf_r (5′-GAAGAAGGGCGAGACCAACA-3′).

### 3.3 Selection of the molecular biomarkers specific for *M. symbioticum* SB0023/3

**Figure 3:**
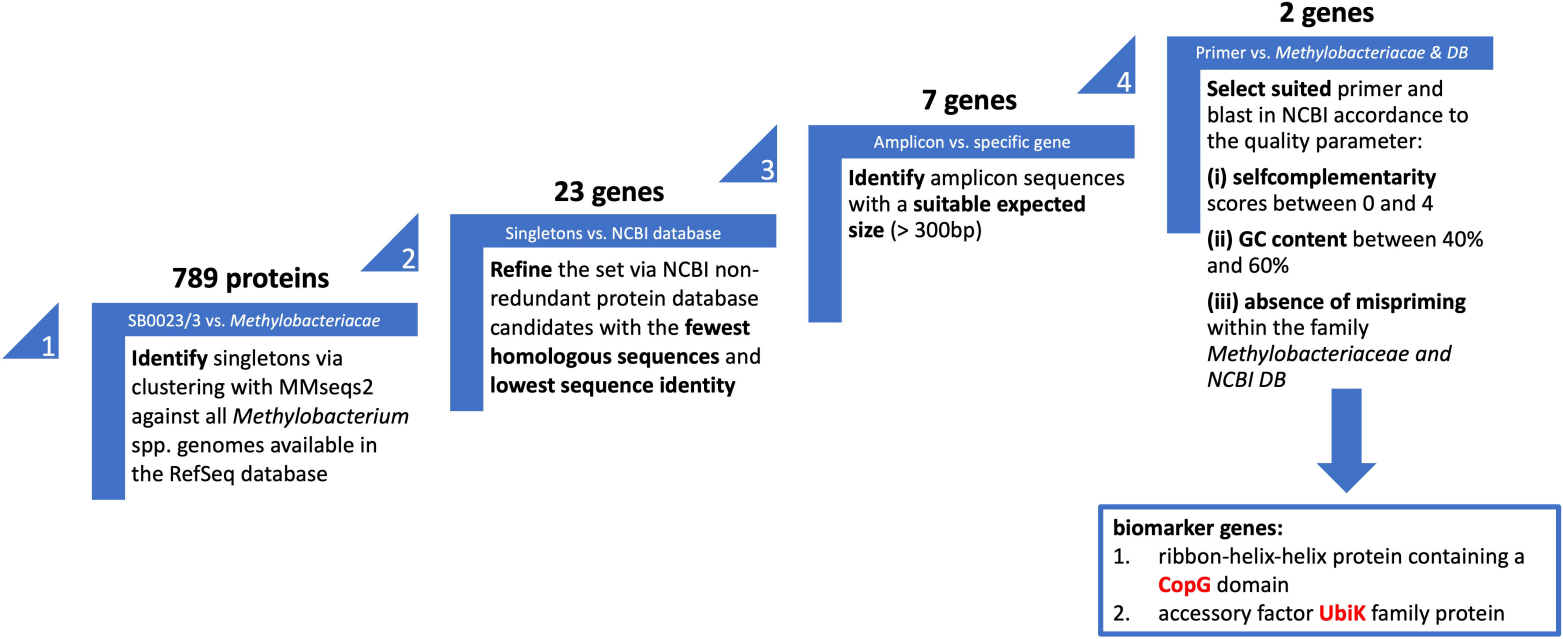
Schematic representation of the stepwise selection process for molecular markers. The name of each selection level is indicated in white lettering within the corresponding step. The criteria applied and data sources used at each stage are listed below the respective level. The output of each selection step, shown above the corresponding level, served as the input for the subsequent step. The outcome of the selection process is presented in the blue box.

To identify a set of potential unique molecular markers specific for *M. symbioticum*, all *Methylobacterium* spp. genomes available in the RefSeq database as of August 2^nd^, 2024, were downloaded. Their protein sequences were subjected to clustering with MMseqs2. As a result, 789 proteins encoded by the resequenced SB0023/3 strain were classified as singletons (**Figure 3**; step 1). To further refine this set, these proteins were queried against the NCBI non-redundant protein database, and candidates with the fewest homologous sequences and the lowest sequence identity were retained. After manual curation, this step yielded 23 genes with predicted functions (**Figure 3**; step 2). Genes annotated as hypothetical proteins were excluded from subsequent analyses.

The identified genes ranged in length from 93 bp to 1,176 bp. Genes shorter than 300 bp (16 in total) were deemed unsuitable due to the expected small amplicon size or questionable annotation (**Figure 3**; step 3). Primer-BLAST was then performed in NCBI for the remaining seven sequences. The resulting primer pairs were evaluated according to the following quality criteria: (i) self-complementarity scores between 0 and 4, and (ii) GC content between 40% and 60%. An additional requirement was the absence of mispriming within the family *Methylobacteriaceae*, which could otherwise produce comparably long PCR products (**Figure 3**; step 4).

After applying these selection criteria, two biomarker genes were retained. The first encodes a ribbon-helix-helix protein containing a CopG domain (contig_1240), and the second encodes an accessory factor UbiK family protein (contig_25530). The following primer pairs were designed GoBio_CopG_f (5′-TCATCACCCAAGCCAACCAG-3′)/GoBio_CopG_r (5′-TCATGGTCGATCCGTCCTCT-3′); GoBio_Ubik_f (5′-GTTCATCGCCCTTGAGGTAG-3′)/GoBio_Ubik_r (5′-GTTCCTTGCGAATGCGGG-3′).

### 3.4 Validation of the selected molecular markers and optimization of the real-time PCR methodology

**Figure 4:**
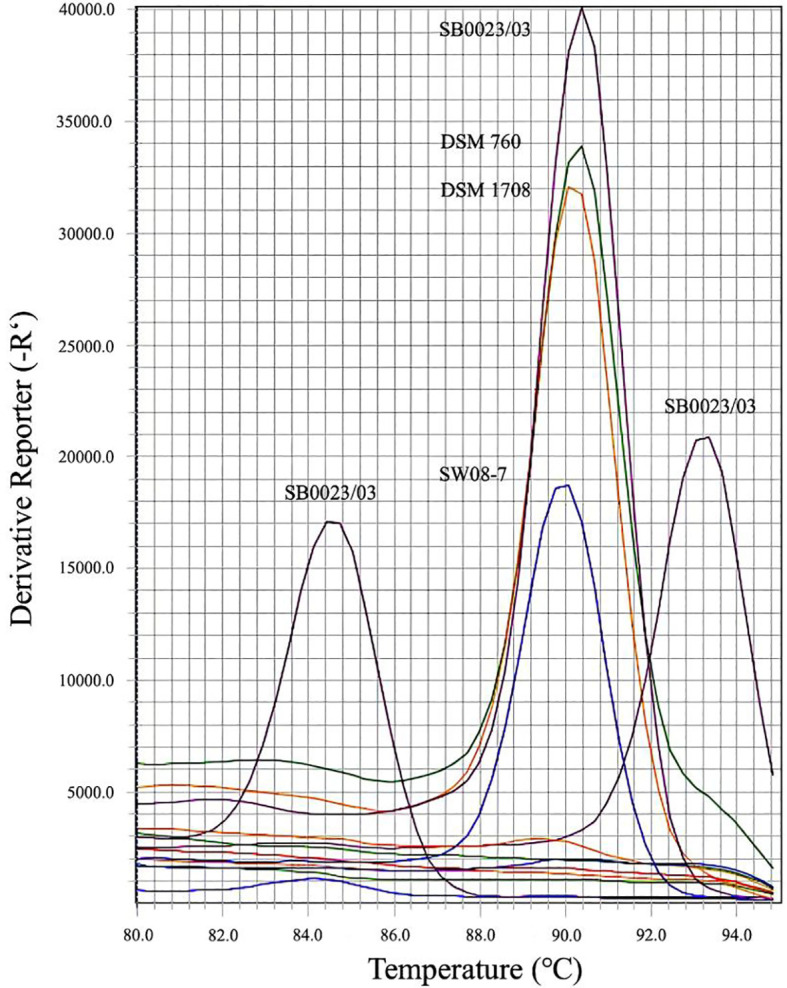
Derivative melting curves for SYBR Green for the *xoxF*-targeting primer (at 90.3 °C) and for the primers targeting *copG* and *ubik* at 84.6 °C and 93.2 °C, respectively. Each peak represents one of the analyzed bacteria. The respective *Methylobacterium* species are indicated next to the peak. These are: *M. symbioticum* SB0023/3, *M. radiotolerans* DSM 760, *M. mesophilicum* DSM 1708 and *M. dankookense* SW08-7.

The specificity of the selected molecular markers (*xoxF*, *copG*, and *ubik*) and PCR primers for biomarker-specific real-time-PCR-based identification was assessed using three reference species – *M. radiotolerans* DSM 760, *M. mesophilicum* DSM 1708, and *M. dankookense* SW08-7 – alongside *M. symbioticum* SB0023/3. The primer pairs GoBio_CopG_f/_r and GoBio_UbiK_f/_r demonstrated high specificity, yielding amplification exclusively in *M. symbioticum* (**Figure 4**). In contrast, the GoBio_XoxF_f/_r primers were non-specific, producing amplification across all four species (**Figure 4**). Melting curve analysis (**Figure 4**) revealed that GoBio_CopG_f/_r and GoBio_UbiK_f/_r produced single, sharp melting peaks at 84.6 °C and 93.2 °C, respectively, exclusively in *M. symbioticum* SB0023/3. By comparison, the non-specific GoBio_XoxF_f/_r primers generated a melting peak at 90.3 °C in all tested species. The gradual temperature increase during the creation of the melting curve was carried out in 0.5 °C increments. This means that the measurement uncertainty of the method is 0.5 °C.

Genomic DNA concentrations were quantified using a SpectraDrop Microplate in a SpectraMax iD3 spectrophotometer (Molecular Devices, San Jose, USA) and standardized to 100 ng per reaction to ensure comparability among PCR samples. Amplification of the *copG* gene in *M. symbioticum* was detected at a cycle threshold (Ct) value of 20, whereas the *ubiK* gene was amplified at a Ct value of 11. The *xoxF*-specific primers amplified DNA from *M. symbioticum* (Ct = 21), *M. dankookense* (Ct = 22), *M. mesophilicum* DSM 1708 (Ct =30), and *M. radiotolerans* (Ct = 32).

Fluorescence intensities of the melt peaks further supported these findings. GoBio_CopG_f/_r and GoBio_UbiK_f/_r produced melt peaks reaching approximately 17,000 and 21,000 fluorescence units, respectively, specific to *M. symbioticum*. For GoBio_XoxF_f/_r, melting peak intensity varied among species: *M. symbioticum* exhibited the highest signal (40,000), followed by *M. radiotolerans* (34,000), *M. mesophilicum* (32,000), and *M. dankookense* (19,000).

### 3.5 Proof-of-concept for monitoring plant colonization success with *M. symbioticum* SB0023/3 using a real-time-PCR-based method of *xoxF*, *copG*, and *ubik* gene detection under controlled conditions

**Figure 5:**
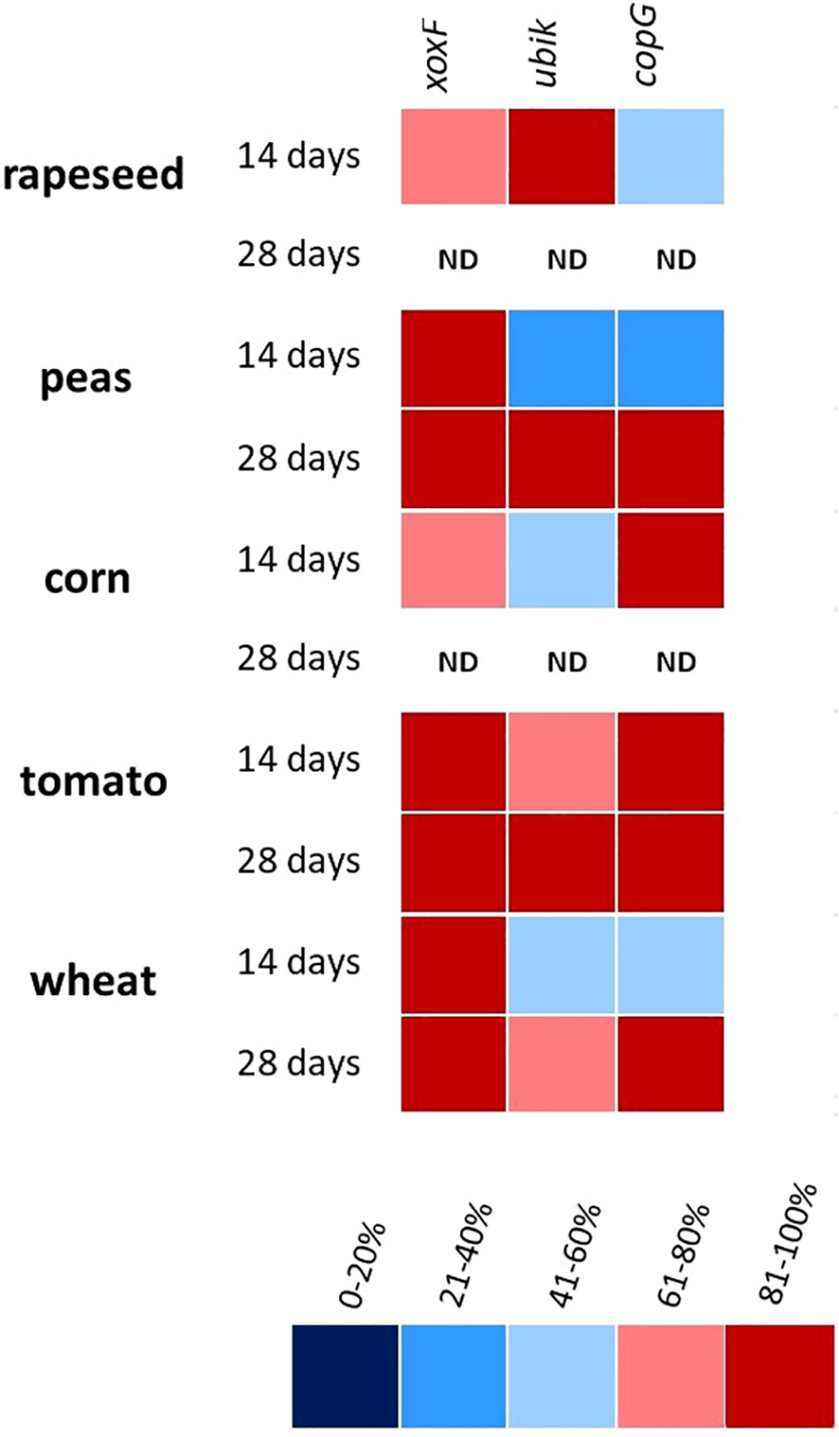
Heatmap showing the plant colonization success after inoculation with *M. symbioticum* SB0023/3 at 14 and 28 days after inoculation. Red colors indicate a higher percentage of positive PCR results and blue colors indicate an increasing percentage of negative PCR results. Columns indicating the respective primer pair that was used for PCR reaction (*xoxF* – GoBio_XoxF_f/_r; *copG* – GoBio_CopG_f/_r; *ubik* – GoBio_Ubik_f/_r. ND indicates “no data” for situations in which samples could not be investigated due to rapid growth of plants outgrowing the space in the climate chamber (as described in the Materials and methods section).

In the next stage of the study, all three primer pairs specific to *xoxF*, *copG*, and *ubik* gene, respectively, were employed to evaluate the success of colonization with *M. symbioticum* SB0023/3 in various arable crops. This experiment served as a proof-of-concept for their application under plant cultivation conditions, rather than solely in pure bacterial cultures. Successful endophytic colonization by *M. symbioticum* SB0023/3 was confirmed in all inoculated plant species (wheat, corn, oilseed rape, pea, and tomato), whereas no bacteria were detected in the non-inoculated controls (**Figure 5**). The real-time PCR analysis of leaf tissues yielded positive amplification with at least one of the three primer pairs (GoBio_CopG_f/_r, GoBio_UbiK_f/_r, or GoBio_XoxF_f/_r) in ≥50% of the samples at both sampling time points (14 and 28 days post-inoculation). The only exception was observed for the GoBio_UbiK_f/_r primers in pea plants at 14 days post-inoculation, where only 30% of the samples tested positive.

For three crops (pea, tomato, and wheat), where samples were analyzed 28 days post-inoculation, the frequency of positive real-time PCR results increased compared to the earlier (14 days) time point. No obvious differences in colonization frequency were detected among the crop species tested. The highest colonization success was observed in tomato plants, where positive detection rates consistently exceeded 80%.

To complement the molecular approach, bacterial re-isolation was performed from leaf extracts using a specially designed selective Cf-Nf medium supplemented with methanol. This classical culturing method additionally confirmed successful colonization by *M. symbioticum* SB0023/3.

## 4 Discussion

The resequencing and comprehensive analysis of the *M. symbioticum* SB0023/3 genome presented in this study substantially expands our understanding of the genetic composition and variability of this plant-associated bacterium, widely used as a biostimulant. Compared with the previously assembled genome (GenBank acc. no. GCF_902141845.1), the resequenced genome revealed notable structural and functional differences, including 121 novel genomic regions (88,574 bp in total) comprising 165 protein-coding genes and five tRNA.

The genomic discrepancies observed between the two assemblies may reflect biological variability among sequenced clones, technical differences in sequencing platforms or assembly pipelines (Rhie et al., 2021), or potential evolutionary events (O’Donnell et al., 2023). Of particular interest is the abundance of mobile genetic elements within the SB0023/3 genome. Several contigs displayed elevated sequencing depth and a hallmark often associated with extrachromosomal elements. Annotation and geNomad analysis confirmed that these contigs correspond to complete bacteriophage genomes, with terminal repeats characteristic of active phage forms (Camargo et al., 2024). This indicates a dynamic phage-host interaction, which may have contributed to genome plasticity in *M. symbioticum* SB0023/3 (Bobay et al., 2013; Piel et al., 2022). In addition, two plasmids were detected. Such elements may facilitate horizontal gene transfer, potentially influencing host adaptation and niche specialization (Dziewit et al., 2014; diCenzo et al., 2016; Lu et al., 2019).

Functionally, the annotated genome contains the typical complement of protein-coding genes and RNA elements expected in *Methylobacterium* spp (Jirakkakul et al., 2023). Clustering into COG categories revealed a distribution consistent with other soil- and phyllosphere-associated bacteria, with approximately 30% of genes assigned to metabolic processes (Carbonetto et al., 2014). A relatively high fraction of hypothetical proteins (~10%) and proteins with domains of unknown function underscores the extent of uncharacterized genetic content and highlights opportunities for novel functional insights.

Importantly, this study addressed the need for reliable molecular tools to specifically detect and monitor *M. symbioticum* SB0023/3. Experimental validation of three primer sets confirmed their utility. The GoBio_CopG_f/_r and GoBio_UbiK_f/_r primers exhibited high specificity, amplifying products exclusively in *M. symbioticum*, whereas the GoBio_XoxF_f/_r primers, targeting the *xoxF* gene – widely present and even duplicated across methylobacterial species (Beck et al., 2015; Taubert et al., 2015) – showed relaxed specificity. Melt curve analysis corroborated these results, differentiating *M. symbioticum* SB0023/3 from *M. radiotolerans*, *M. mesophilicum*, and *M. dankookense* when using GoBio_CopG_f/_r and GoBio_UbiK_f/_r. Notably, the absence of amplification in *M. dankookense*, despite its close phylogenetic relationship (Pascual et al., 2020; Leducq et al., 2022), further emphasizes the specificity of these markers. This highlights the potential of the molecular markers developed in comparison to other identification methods. For example, it is not possible to clearly distinguish between M. *symbioticum* and M. *dankookense* via 16S rRNA analysis. The threshold value for reliable species identification is typically given as approximately 98.65-99% (Kim et al., 2014; Rodriguez-R et al., 2018; Hackmann, 2025). The 16S rRNA identity between *M. symbioticum* and *M. dankookense* is 98.7%. Differences were also reflected in Ct values for GoBio_XoxF_f/_r, with *M. symbioticum* amplifying earlier and with higher fluorescence intensity, consistent with reduced template matching and specificity in non-SB0023/3 strains. Nevertheless, GoBio_XoxF_f/_r alone cannot be recommended for reliable detection of *M. symbioticum* SB0023/3.

A proof-of-concept under controlled conditions for the developed detection system was provided by tracking the colonization efficiency of various crop plants at 14- and 28-day post-inoculation. The results demonstrated successful endophytic colonization of pea, maize, tomato, wheat, and oilseed species by *M. symbioticum*. The real-time-PCR-based method enabled direct detection of colonization from leaf tissues without the need for prior bacterial cultivation on selective media.

**Figure 6:**
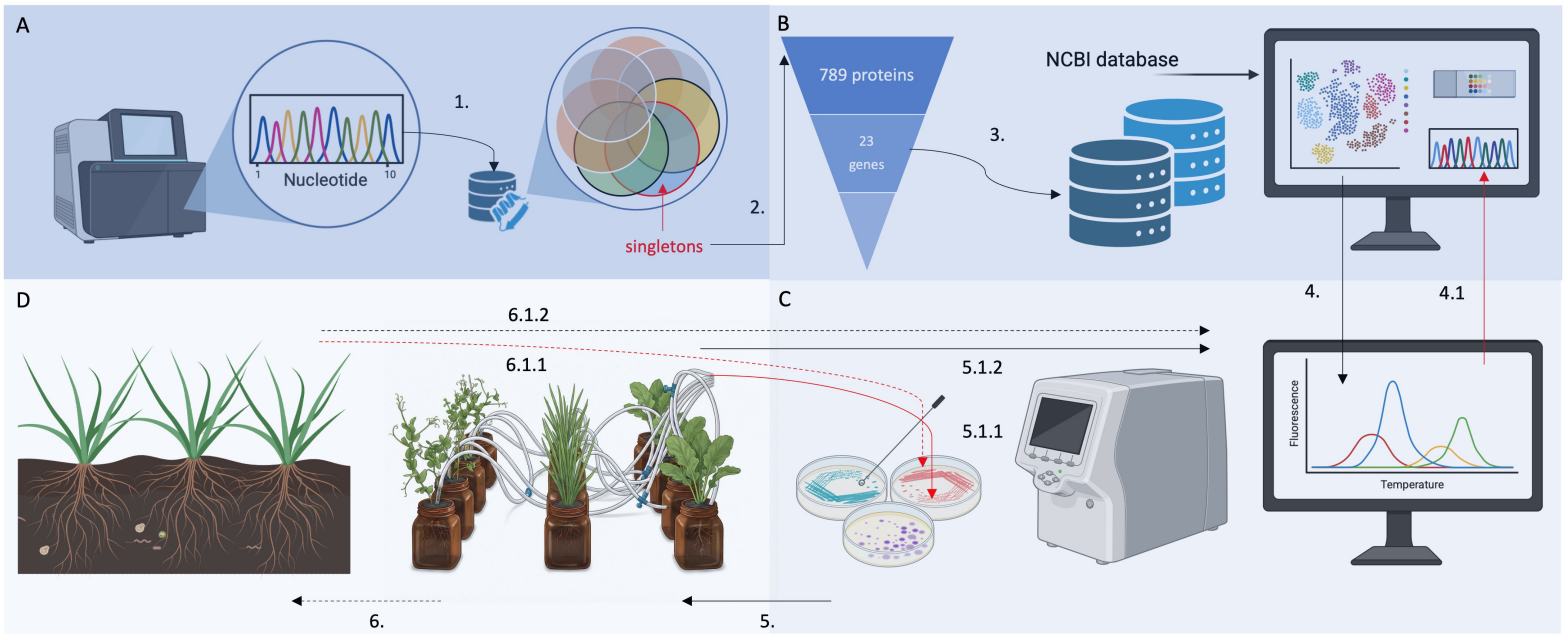
Schematic representation of the proposed pipeline of biomonitoring. **(A)** whole genome sequencing of the target strain and subsequent search for singletons by comparison with protein database (step 1). **(B)** Refining the set of potential biomarkers (based on singleton analysis) via the NCBI non-redundant protein database comparisons (steps 2 and 3) to find candidates with the fewest homologous sequences. Testing the potential primers via *in silico* PCR and checking primer quality parameters. **(C)***In vitro* PCR application of the selected primers (step 4) in both the target strain and closely related, reference strains. **(D)** Proof-of-concept under controlled (step 5) and field conditions (step 6) via PCR analysis directly from plant material (step 5.1.2 and 6.1.2) and (step 5.1.1 and 6.1.1) using material from cultivation on selective culture media (step 5.1.1 and 6.1.1). Red arrows (step 5.1.1, 6.1.1, and 4.1) indicate a second line of evidence. Step 4.1 represents a reinsurance by sequencing the PCR amplicon and comparing it to the estimated sequence. The arrows with dotted lines refer to analyses (field study) whose results are not presented in this paper and will be published at a later date, and were presented here to show the complete pipeline and broad perspective of the study.

Microbial complexity under real-life conditions (e.g., soil, rhizosphere, phyllosphere) may negatively influence the sensitivity, specificity, and quantitative accuracy of molecular detection. Nevertheless, real-time PCR assays can operate even using extremely low DNA concentrations in complex environments, giving the chance of detecting microorganisms occurring in minorities. To ensure accuracy, careful primer-focused assay design and testing against closely related species are essential to avoid misleading false positives and negatives (Wilcox et al., 2013). Taking these principles into account, we have placed great emphasis on the quality parameters of the primers used (**Figures 3**, **6**) and on validating them against the NCBI database resources and ensuring their selectivity in comparison to the closest known related species. Although databases are becoming increasingly comprehensive, and *in silico* testing of primers can provide a more holistic approach than *in vitro* testing could ever achieve, cultivation remains a valuable complementary approach. Since bacterial DNA may persist in plant tissues after cell death, PCR-based assays cannot determine bacterial viability at the time of sampling. For this reason, we recommend a combined application of cultivation and molecular techniques, as demonstrated in this study. Here, plating was performed using the same plant extracts analyzed by real-time PCR, allowing direct comparison of results while minimizing methodological variability (**Figure 6**).

However, cultivation is not recommended as a routine preliminary step for three main reasons. First, isolation of viable bacteria requires advanced methodology during sampling, preparation of extracts, and transport – often a significant challenge under field conditions. Second, rigorous surface sterilization necessary for eliminating epiphytic bacterial contaminants may inadvertently reduce endophyte viability. Third, despite selective conditions targeting diazotrophs and methylotrophs, non-target organisms may proliferate, sometimes exploiting *Methylobacterium* spp. as pioneer colonizers providing limiting nutrients (Torres et al., 2022).

This study demonstrated that by combining one non-specific primer set (GoBio_XoxF_f/_r) with two highly specific sets (GoBio_CopG_f/_r and GoBio_UbiK_f/_r), a robust strategy was established to distinguish between naturally occurring methylobacterial colonization (by indigenous soil or phyllosphere strains) and deliberate inoculation with *M. symbioticum* SB0023/3. The development of a simple, rapid, and reliable detection system is important not only from a biosafety perspective – for instance, monitoring unintended spread and potential impacts on soil microbial biodiversity – but also for practical field applications. Well-validated molecular biomarkers provide a valuable tool for tracing bacterial dispersal dynamics and investigating potential mechanisms of transmission into untreated areas. For *M. symbioticum* SB0023/3 in particular, accurate detection methods are critically needed, as the strain is widely used as a biostimulant in North and South America, Europe, Asia, and Australia. Reliable confirmation of colonization is essential to differentiate between genuine biological inefficacy and technical failures during application or cultivation that may result in unsuccessful inoculation (Beirne, 1985; Knights et al., 2021). Robust detection systems, combined with a clear understanding of colonization mechanisms, are therefore essential for accurately diagnosing the underlying causes of failed inoculation/colonization events.

The detection system developed in this study was evaluated under controlled laboratory conditions, with only preliminary field trials conducted to date. Although the initial field results were encouraging, further rounds of testing are required and are constrained by vegetation cycles. Given the extended time frame necessary to complete comprehensive field validation, we have chosen to present the method to the scientific community at this stage. In the spirit of open scientific evaluation, we anticipate that early dissemination will facilitate broader and more rigorous validation through independent field studies, alongside our ongoing investigations.

Nevertheless, several challenges should be considered when evaluating the system under field conditions. The principal limitations include: (i) natural microbial complexity, which may influence primer performance – although the formation of non-specific amplification products is considered unlikely due to comprehensive *in silico* validation against the NCBI database, a more relevant concern is the effective titration of primers through non-specific binding to abundant background (environmental) DNA; (ii) inhibition of amplification by naturally occurring PCR inhibitors that may be present in environmental samples; and (iii) technical constraints related to experimental design, including the number of samples required to achieve statistically robust outcomes under heterogeneous field conditions.

In addition, general limitations associated with the use of molecular markers in biomonitoring, particularly in biosafety-related applications, must be acknowledged. PCR-based approaches detect DNA irrespective of cell viability; therefore, the presence of a molecular signal does not necessarily indicate viable or metabolically active cells. This may lead to an overestimation of persistence or colonization in environmental samples. Furthermore, DNA-based markers provide limited insight into the physiological state or functional activity of the detected organism. As a result, detection confirms presence but does not directly demonstrate metabolic activity or ecological impact.

It is also important to consider general limitations arising from the applied experimental framework (**Figure 6**), particularly from a future biomonitoring perspective. First, the presence of mobile genetic elements in any bacterial strain intended for monitoring represents an important factor influencing long-term assay robustness. In the present study, the selected biomarkers (*copG* and *ubik*) are chromosomally encoded, which reduces the risk of target loss during cultivation and minimizes variability in copy number between cells. However, when designing biomarkers for tracking biofertilizers or biostimulants more broadly, it is essential to determine whether candidate loci are located on mobile elements such as plasmids, prophages, or transposon-associated regions. Markers residing on such elements may be horizontally transferred to other bacteria, potentially leading to false-positive signals in field monitoring and complicating biosafety assessments. Furthermore, mobile genetic elements often occur in multiple copies and may exhibit variable copy numbers across populations or environmental conditions (e.g., episomal versus integrated prophage states). This variability can artificially elevate qPCR signals and compromise longitudinal comparability of quantitative data. Therefore, future biomarker development pipelines should explicitly include replicon-level localization of target genes (chromosome vs. plasmid/phage), evaluation of copy-number stability, and validation using field reisolates as well as different production batches. Second, while this proof-of-concept focused on leaf tissues, the same marker framework can be adapted to other matrices relevant for agricultural monitoring (e.g., seed coats, rhizosphere/soil, or root tissues), but such extensions require matrix-specific optimization due to increased microbial complexity and PCR inhibitors in soil, as well as the higher likelihood of detecting extracellular DNA. Finally, false positives and false negatives have distinct consequences in field monitoring: false positives could lead to overestimation of inoculant persistence, whereas false negatives could mask successful colonization and confound interpretation of performance trials. To mitigate these risks, a conservative interpretation strategy is recommended, including strict assay thresholds, use of multiple independent markers (as implemented in this study), appropriate inhibition controls, and – where decisions depend on viability – complementary cultivation-based verification or viability-informed molecular approaches.

In summary, the results presented here establish a foundation for the molecular characterization of *M. symbioticum* SB0023/3 and highlight the potential of genome-informed biomarker design in environmental monitoring and agricultural biotechnology. The ability to detect *M. symbioticum* SB0023/3 directly from plant tissues without cultivation greatly facilitates studies on colonization dynamics, ecological interactions, and biosafety. Moreover, the experimental approach described here could be extended (as a pipeline) to other microbial strains used as biostimulants or biocontrol agents, supporting their responsible and traceable application in sustainable agriculture (**Figure 6**).

## Data Availability

The datasets presented in this study can be found in online repositories. The names of the repository/repositories and accession number(s) can be found below: https://www.ncbi.nlm.nih.gov/genbank/, PRJNA1330767.
