# Supplementary material for: Development of molecular biomarkers for monitoring of arable crops colonization with Methylobacterium symbioticum SB0023/3, a methylotrophic bacterium commonly used as a biostimulant in agriculture
Source: Front Plant Sci. 2026 Mar 2;17:1718185. doi: 10.3389/fpls.2026.1718185 (PMC12989545; doi:10.3389/fpls.2026.1718185)
Supplement: Supplementary file 1 [file Table1.docx]

- Supplemenatary material -

Development of molecular biomarkers for monitoring of arable crops colonisation with *Methylobacterium symbioticum* SB0023/3, a methylotrophic bacterium commonly used as a biostimulant in agriculture

Rolf Allner^1,2^, Przemyslaw Decewicz^2^, Thomas Allner^1^, Agata Bluszcz^2^, Lukasz Dziewit^2*^

^1^GOBIO GmbH - Institute for Aquatic Ecology and Applied Biology, Aarbergen, Germany

^2^Laboratory of Applied Microbial Ecology, Institute of Bioengineering, Faculty of Biology, University of Warsaw, Warsaw, Poland

*** Correspondence:**Lukasz Dziewit
l.dziewit@uw.edu.pl

**Supplementary Table 1.** The list and coordinates of features annotated within the DNA regions (at least 10 bp) of the resequenced genome that lacked significant similarity (e-value > 1e−10) to the previously deposited genome of *M. symbioticum* SB0023/3 (GenBank acc. no. GCF_902141845.1).

| **Feature type** | **Locus tag** | **Feature start** | **Feature end** | **Strand** | **CDS product** |
| --- | --- | --- | --- | --- | --- |
| gene | JPFDDB_00005 | 226 | 718 | 1 |  |
| CDS | JPFDDB_00005 | 226 | 718 | 1 | chemotaxis protein CheW |
| gene | JPFDDB_00010 | 731 | 2273 | 1 |  |
| CDS | JPFDDB_00010 | 731 | 2273 | 1 | protein-glutamate O-methyltransferase CheR |
| gene | JPFDDB_00015 | 2269 | 2821 | 1 |  |
| CDS | JPFDDB_00015 | 2269 | 2821 | 1 | chemotaxis protein CheW |
| gene | JPFDDB_00020 | 2874 | 4545 | 1 |  |
| CDS | JPFDDB_00020 | 2874 | 4545 | 1 | Frizzy aggregation protein FrzCD |
| gene | JPFDDB_00025 | 4564 | 6856 | 1 |  |
| CDS | JPFDDB_00025 | 4564 | 6856 | 1 | response regulator |
| gene | JPFDDB_00560 | 103973 | 106100 | -1 |  |
| CDS | JPFDDB_00560 | 103973 | 106100 | -1 | pseudouridine synthase |
| gene | JPFDDB_01570 | 316748 | 318200 | 1 |  |
| CDS | JPFDDB_01570 | 316748 | 318200 | 1 | ATP-dependent RNA helicase RhlE |
| gene | JPFDDB_01730 | 352393 | 353896 | -1 |  |
| CDS | JPFDDB_01730 | 352393 | 353896 | -1 | Peptidase M15A C-terminal domain-containing protein |
| gene | JPFDDB_02045 | 430334 | 431711 | -1 |  |
| CDS | JPFDDB_02045 | 430334 | 431711 | -1 | signal recognition particle-docking protein FtsY |
| gene | JPFDDB_02060 | 434913 | 436320 | -1 |  |
| CDS | JPFDDB_02060 | 434913 | 436320 | -1 | DUF2336 domain-containing protein |
| gene | JPFDDB_02065 | 436316 | 437372 | -1 |  |
| CDS | JPFDDB_02065 | 436316 | 437372 | -1 | Transglycosylase SLT domain-containing protein |
| gene | JPFDDB_02070 | 437477 | 438122 | 1 |  |
| CDS | JPFDDB_02070 | 437477 | 438122 | 1 | aldolase/citrate lyase family protein |
| gene | JPFDDB_02215 | 466960 | 469933 | 1 |  |
| CDS | JPFDDB_02215 | 466960 | 469933 | 1 | translation initiation factor IF-2 |
| gene | JPFDDB_02270 | 482780 | 483791 | -1 |  |
| CDS | JPFDDB_02270 | 482780 | 483791 | -1 | tRNA-dihydrouridine synthase |
| gene | JPFDDB_02580 | 549234 | 551502 | -1 |  |
| CDS | JPFDDB_02580 | 549234 | 551502 | -1 | hypothetical protein |
| gene | JPFDDB_02590 | 552899 | 556337 | 1 |  |
| CDS | JPFDDB_02590 | 552899 | 556337 | 1 | DNA replication and repair protein RecF |
| gene | JPFDDB_02755 | 592125 | 593304 | -1 |  |
| CDS | JPFDDB_02755 | 592125 | 593304 | -1 | Periplasmic serine endoprotease DegP |
| gene | JPFDDB_02765 | 594278 | 597713 | -1 |  |
| CDS | JPFDDB_02765 | 594278 | 597713 | -1 | Chromosome partition protein Smc |
| gene | JPFDDB_03015 | 655556 | 656708 | -1 |  |
| CDS | JPFDDB_03015 | 655556 | 656708 | -1 | Mannosyl-glycoprotein endo-beta-N-acetylglucosamidase-like domain-containing protein |
| gene | JPFDDB_03620 | 782341 | 782620 | 1 |  |
| CDS | JPFDDB_03620 | 782341 | 782620 | 1 | Glycine zipper domain-containing protein |
| gene | JPFDDB_03870 | 838978 | 840871 | -1 |  |
| CDS | JPFDDB_03870 | 838978 | 840871 | -1 | Putative peptidoglycan D,D-transpeptidase FtsI |
| gene | JPFDDB_04690 | 1004737 | 1006648 | 1 |  |
| CDS | JPFDDB_04690 | 1004737 | 1006648 | 1 | DNA polymerase III subunit gamma/tau |
| gene | JPFDDB_06580 | 1432584 | 1435185 | 1 |  |
| CDS | JPFDDB_06580 | 1432584 | 1435185 | 1 | Chemotaxis protein CheA |
| gene | JPFDDB_06925 | 1528209 | 1529040 | -1 |  |
| CDS | JPFDDB_06925 | 1528209 | 1529040 | -1 | hypothetical protein |
| gene | JPFDDB_07745 | 1703809 | 1706269 | 1 |  |
| CDS | JPFDDB_07745 | 1703809 | 1706269 | 1 | ATP-dependent helicase HrpB |
| gene | JPFDDB_07770 | 1709228 | 1710452 | 1 |  |
| CDS | JPFDDB_07770 | 1709228 | 1710452 | 1 | CCA-adding enzyme |
| gene | JPFDDB_08435 | 1852151 | 1854065 | -1 |  |
| CDS | JPFDDB_08435 | 1852151 | 1854065 | -1 | glycosyltransferase |
| gene | JPFDDB_08930 | 1952263 | 1952746 | -1 |  |
| CDS | JPFDDB_08930 | 1952263 | 1952746 | -1 | Permease |
| gene | JPFDDB_09030 | 1973771 | 1974977 | -1 |  |
| CDS | JPFDDB_09030 | 1973771 | 1974977 | -1 | Protein-glutamate methylesterase/protein-glutamine glutaminase |
| gene | JPFDDB_09200 | 2005921 | 2006218 | -1 |  |
| CDS | JPFDDB_09200 | 2005921 | 2006218 | -1 | Heat shock protein 15 |
| gene | JPFDDB_09205 | 2006234 | 2009546 | -1 |  |
| CDS | JPFDDB_09205 | 2006234 | 2009546 | -1 | Helicase HelY |
| gene | JPFDDB_09740 | 2125123 | 2126158 | -1 |  |
| CDS | JPFDDB_09740 | 2125123 | 2126158 | -1 | KipI antagonist |
| gene | JPFDDB_09745 | 2126150 | 2126894 | -1 |  |
| CDS | JPFDDB_09745 | 2126150 | 2126894 | -1 | 5-oxoprolinase subunit PxpB |
| gene | JPFDDB_10155 | 2220865 | 2220942 | -1 |  |
| tRNA | JPFDDB_10155 | 2220865 | 2220942 | -1 | tRNA-Ile(gat) |
| gene | JPFDDB_10225 | 2240457 | 2243658 | -1 |  |
| CDS | JPFDDB_10225 | 2240457 | 2243658 | -1 | double-strand break repair protein AddB |
| gene | JPFDDB_10490 | 2294211 | 2295186 | -1 |  |
| CDS | JPFDDB_10490 | 2294211 | 2295186 | -1 | Aliphatic sulfonates-binding protein |
| gene | JPFDDB_10495 | 2295182 | 2295773 | -1 |  |
| CDS | JPFDDB_10495 | 2295182 | 2295773 | -1 | FMN reductase |
| gene | JPFDDB_10610 | 2319150 | 2320476 | 1 |  |
| CDS | JPFDDB_10610 | 2319150 | 2320476 | 1 | Pseudooxynicotine oxidase |
| gene | JPFDDB_10650 | 2326510 | 2327533 | -1 |  |
| CDS | JPFDDB_10650 | 2326510 | 2327533 | -1 | threonine-phosphate decarboxylase CobD |
| gene | JPFDDB_10745 | 2345491 | 2345920 | 1 |  |
| CDS | JPFDDB_10745 | 2345491 | 2345920 | 1 | Cobalt-precorrin-5A hydrolase |
| gene | JPFDDB_10850 | 2370928 | 2372386 | -1 |  |
| CDS | JPFDDB_10850 | 2370928 | 2372386 | -1 | type VI secretion system-associated FHA domain protein TagH |
| gene | JPFDDB_10870 | 2375644 | 2377027 | 1 |  |
| CDS | JPFDDB_10870 | 2375644 | 2377027 | 1 | type VI secretion system protein TssA |
| gene | JPFDDB_10920 | 2389600 | 2393488 | 1 |  |
| CDS | JPFDDB_10920 | 2389600 | 2393488 | 1 | Heme/hemopexin-binding protein |
| gene | JPFDDB_10930 | 2395493 | 2396324 | -1 |  |
| CDS | JPFDDB_10930 | 2395493 | 2396324 | -1 | hypothetical protein |
| gene | JPFDDB_11055 | 2429258 | 2430260 | 1 |  |
| CDS | JPFDDB_11055 | 2429258 | 2430260 | 1 | Putative NTE family protein |
| gene | JPFDDB_11060 | 2430241 | 2431435 | -1 |  |
| CDS | JPFDDB_11060 | 2430241 | 2431435 | -1 | N-acetylglucosamine-6-phosphate deacetylase |
| gene | JPFDDB_11065 | 2431431 | 2432430 | -1 |  |
| CDS | JPFDDB_11065 | 2431431 | 2432430 | -1 | Glutamine--fructose-6-phosphate aminotransferase [isomerizing] |
| gene | JPFDDB_11155 | 2451575 | 2452097 | 1 |  |
| CDS | JPFDDB_11155 | 2451575 | 2452097 | 1 | Regulatory protein RecX |
| gene | JPFDDB_11320 | 2482932 | 2483922 | -1 |  |
| CDS | JPFDDB_11320 | 2482932 | 2483922 | -1 | DUF1311 domain-containing protein |
| gene | JPFDDB_11360 | 2491828 | 2492614 | 1 |  |
| CDS | JPFDDB_11360 | 2491828 | 2492614 | 1 | Integral membrane protein TerC |
| gene | JPFDDB_11365 | 2492662 | 2493049 | -1 |  |
| CDS | JPFDDB_11365 | 2492662 | 2493049 | -1 | DNA-binding protein |
| gene | JPFDDB_11975 | 2604004 | 2605525 | 1 |  |
| CDS | JPFDDB_11975 | 2604004 | 2605525 | 1 | putative periplasmic serine endoprotease DegP-like |
| gene | JPFDDB_12010 | 2614479 | 2615682 | -1 |  |
| CDS | JPFDDB_12010 | 2614479 | 2615682 | -1 | Multidrug resistance protein MdtE |
| gene | JPFDDB_12760 | 2776838 | 2778311 | -1 |  |
| CDS | JPFDDB_12760 | 2776838 | 2778311 | -1 | Cytochrome c-type biogenesis protein CycH |
| gene | JPFDDB_13210 | 2863887 | 2865615 | -1 |  |
| CDS | JPFDDB_13210 | 2863887 | 2865615 | -1 | AsmA-like C-terminal region-containing protein |
| gene | JPFDDB_13230 | 2870662 | 2870739 | 1 |  |
| tRNA | JPFDDB_13230 | 2870662 | 2870739 | 1 | tRNA-Ile(gat) |
| gene | JPFDDB_13285 | 2883606 | 2886561 | 1 |  |
| CDS | JPFDDB_13285 | 2883606 | 2886561 | 1 | Ribonuclease E |
| gene | JPFDDB_13795 | 3001246 | 3001768 | -1 |  |
| CDS | JPFDDB_13795 | 3001246 | 3001768 | -1 | Glycosyltransferase 2-like domain-containing protein |
| gene | JPFDDB_14145 | 3076438 | 3077311 | -1 |  |
| CDS | JPFDDB_14145 | 3076438 | 3077311 | -1 | DUF6456 domain-containing protein |
| gene | JPFDDB_14190 | 3086357 | 3089057 | 1 |  |
| CDS | JPFDDB_14190 | 3086357 | 3089057 | 1 | N-acetyltransferase domain-containing protein |
| gene | JPFDDB_14315 | 3108150 | 3111636 | 1 |  |
| CDS | JPFDDB_14315 | 3108150 | 3111636 | 1 | AsmA protein |
| gene | JPFDDB_14820 | 3225052 | 3226222 | -1 |  |
| CDS | JPFDDB_14820 | 3225052 | 3226222 | -1 | Glycosyltransferase 2-like domain-containing protein |
| gene | JPFDDB_14840 | 3231815 | 3232901 | 1 |  |
| CDS | JPFDDB_14840 | 3231815 | 3232901 | 1 | molybdopterin-binding protein |
| gene | JPFDDB_15135 | 3302177 | 3304010 | 1 |  |
| CDS | JPFDDB_15135 | 3302177 | 3304010 | 1 | Flagellar protein |
| gene | JPFDDB_15140 | 3304117 | 3304510 | 1 |  |
| CDS | JPFDDB_15140 | 3304117 | 3304510 | 1 | porin |
| gene | JPFDDB_15185 | 3311021 | 3311747 | -1 |  |
| CDS | JPFDDB_15185 | 3311021 | 3311747 | -1 | Co-chaperone protein DjlA |
| gene | JPFDDB_15190 | 3311925 | 3312195 | -1 |  |
| CDS | JPFDDB_15190 | 3311925 | 3312195 | -1 | Glycine zipper domain-containing protein |
| gene | JPFDDB_15330 | 3335143 | 3342940 | -1 |  |
| CDS | JPFDDB_15330 | 3335143 | 3342940 | -1 | Apolipoprotein acyltransferase |
| gene | JPFDDB_15710 | 3417375 | 3418419 | 1 |  |
| CDS | JPFDDB_15710 | 3417375 | 3418419 | 1 | hypothetical protein |
| gene | JPFDDB_16265 | 3538545 | 3539094 | 1 |  |
| CDS | JPFDDB_16265 | 3538545 | 3539094 | 1 | 5-methylcytosine-specific restriction endonuclease McrA |
| gene | JPFDDB_16270 | 3539101 | 3539362 | -1 |  |
| CDS | JPFDDB_16270 | 3539101 | 3539362 | -1 | hypothetical protein |
| gene | JPFDDB_16370 | 3558676 | 3561208 | -1 |  |
| CDS | JPFDDB_16370 | 3558676 | 3561208 | -1 | ABC transporter ATP-binding protein |
| gene | JPFDDB_16420 | 3570041 | 3571652 | -1 |  |
| CDS | JPFDDB_16420 | 3570041 | 3571652 | -1 | LysM peptidoglycan-binding domain-containing protein |
| gene | JPFDDB_16440 | 3575799 | 3577764 | 1 |  |
| CDS | JPFDDB_16440 | 3575799 | 3577764 | 1 | AI-2 transport protein TqsA |
| gene | JPFDDB_16470 | 3582869 | 3583892 | 1 |  |
| CDS | JPFDDB_16470 | 3582869 | 3583892 | 1 | DUF2865 domain-containing protein |
| gene | JPFDDB_16475 | 3584082 | 3586215 | 1 |  |
| CDS | JPFDDB_16475 | 3584082 | 3586215 | 1 | OmpA family protein |
| gene | JPFDDB_16525 | 3596452 | 3596529 | 1 |  |
| tRNA | JPFDDB_16525 | 3596452 | 3596529 | 1 | tRNA-Ile(gat) |
| gene | JPFDDB_16655 | 3624045 | 3627315 | 1 |  |
| CDS | JPFDDB_16655 | 3624045 | 3627315 | 1 | tetratricopeptide repeat protein |
| gene | JPFDDB_17740 | 3842764 | 3844141 | 1 |  |
| CDS | JPFDDB_17740 | 3842764 | 3844141 | 1 | Ribosomal large subunit pseudouridine synthase C |
| gene | JPFDDB_18555 | 4034032 | 4034109 | -1 |  |
| tRNA | JPFDDB_18555 | 4034032 | 4034109 | -1 | tRNA-Ile(gat) |
| gene | JPFDDB_19695 | 4289147 | 4291625 | -1 |  |
| CDS | JPFDDB_19695 | 4289147 | 4291625 | -1 | peptidoglycan glycosyltransferase |
| gene | JPFDDB_20275 | 4409690 | 4412312 | 1 |  |
| CDS | JPFDDB_20275 | 4409690 | 4412312 | 1 | DNA translocase FtsK |
| gene | JPFDDB_20330 | 4428149 | 4429346 | -1 |  |
| CDS | JPFDDB_20330 | 4428149 | 4429346 | -1 | ABC-2 type transporter transmembrane domain-containing protein |
| gene | JPFDDB_20335 | 4429342 | 4430314 | -1 |  |
| CDS | JPFDDB_20335 | 4429342 | 4430314 | -1 | Putative ABC transporter ATP-binding protein YbhF |
| gene | JPFDDB_20430 | 4444420 | 4447240 | 1 |  |
| CDS | JPFDDB_20430 | 4444420 | 4447240 | 1 | Aerotolerance regulator N-terminal domain-containing protein |
| gene | JPFDDB_20745 | 4527021 | 4527621 | -1 |  |
| CDS | JPFDDB_20745 | 4527021 | 4527621 | -1 | hypothetical protein |
| gene | JPFDDB_21185 | 4620025 | 4621372 | 1 |  |
| CDS | JPFDDB_21185 | 4620025 | 4621372 | 1 | SPOR domain-containing protein |
| gene | JPFDDB_21300 | 4641139 | 4642543 | -1 |  |
| CDS | JPFDDB_21300 | 4641139 | 4642543 | -1 | flagellar hook-length control protein FliK |
| gene | JPFDDB_21460 | 4681363 | 4682842 | -1 |  |
| CDS | JPFDDB_21460 | 4681363 | 4682842 | -1 | DNA polymerase Y family protein |
| gene | JPFDDB_21465 | 4682855 | 4683668 | -1 |  |
| CDS | JPFDDB_21465 | 4682855 | 4683668 | -1 | ImuA protein |
| gene | JPFDDB_21470 | 4683903 | 4684743 | 1 |  |
| CDS | JPFDDB_21470 | 4683903 | 4684743 | 1 | collagen-like protein |
| gene | JPFDDB_21475 | 4684870 | 4686202 | -1 |  |
| CDS | JPFDDB_21475 | 4684870 | 4686202 | -1 | H(+)/Cl(-) exchange transporter ClcA |
| gene | JPFDDB_21480 | 4686504 | 4687065 | 1 |  |
| CDS | JPFDDB_21480 | 4686504 | 4687065 | 1 | Beta-Ig-H3/fasciclin |
| gene | JPFDDB_21485 | 4687123 | 4687858 | -1 |  |
| CDS | JPFDDB_21485 | 4687123 | 4687858 | -1 | anti-sigma factor |
| gene | JPFDDB_21525 | 4693847 | 4694732 | 1 |  |
| CDS | JPFDDB_21525 | 4693847 | 4694732 | 1 | Membrane-bound lytic murein transglycosylase C |
| gene | JPFDDB_21725 | 4731215 | 4732727 | -1 |  |
| CDS | JPFDDB_21725 | 4731215 | 4732727 | -1 | Porin B |
| gene | JPFDDB_21925 | 4767900 | 4769937 | 1 |  |
| CDS | JPFDDB_21925 | 4767900 | 4769937 | 1 | p-hydroxybenzoic acid efflux pump subunit AaeB |
| gene | JPFDDB_22190 | 4820960 | 4821359 | 1 |  |
| CDS | JPFDDB_22190 | 4820960 | 4821359 | 1 | hypothetical protein |
| gene | JPFDDB_22195 | 4821478 | 4824898 | 1 |  |
| CDS | JPFDDB_22195 | 4821478 | 4824898 | 1 | Tetratricopeptide repeat protein |
| gene | JPFDDB_22200 | 4825071 | 4826265 | 1 |  |
| CDS | JPFDDB_22200 | 4825071 | 4826265 | 1 | BioF2-like acetyltransferase domain-containing protein |
| gene | JPFDDB_22205 | 4826332 | 4828645 | -1 |  |
| CDS | JPFDDB_22205 | 4826332 | 4828645 | -1 | Polysaccharide chain length determinant N-terminal domain-containing protein |
| gene | JPFDDB_22275 | 4841801 | 4842347 | -1 |  |
| CDS | JPFDDB_22275 | 4841801 | 4842347 | -1 | hypothetical protein |
| gene | JPFDDB_22875 | 4957227 | 4959570 | -1 |  |
| CDS | JPFDDB_22875 | 4957227 | 4959570 | -1 | DUF5682 domain-containing protein |
| gene | JPFDDB_22885 | 4960679 | 4962266 | -1 |  |
| CDS | JPFDDB_22885 | 4960679 | 4962266 | -1 | DUF5691 domain-containing protein |
| gene | JPFDDB_22890 | 4962265 | 4963627 | -1 |  |
| CDS | JPFDDB_22890 | 4962265 | 4963627 | -1 | SWIM-type domain-containing protein |
| gene | JPFDDB_23260 | 5039400 | 5041251 | 1 |  |
| CDS | JPFDDB_23260 | 5039400 | 5041251 | 1 | NADH-quinone oxidoreductase subunit L |
| gene | JPFDDB_23305 | 5050738 | 5051206 | -1 |  |
| CDS | JPFDDB_23305 | 5050738 | 5051206 | -1 | Secreted protein |
| gene | JPFDDB_23405 | 5070502 | 5070931 | 1 |  |
| CDS | JPFDDB_23405 | 5070502 | 5070931 | 1 | Secreted protein |
| gene | JPFDDB_23465 | 5081010 | 5083074 | 1 |  |
| CDS | JPFDDB_23465 | 5081010 | 5083074 | 1 | DNA repair exonuclease SbcCD ATPase subunit |
| gene | JPFDDB_23800 | 5158739 | 5160119 | -1 |  |
| CDS | JPFDDB_23800 | 5158739 | 5160119 | -1 | Metallophosphoesterase |
| gene | JPFDDB_23805 | 5160222 | 5161557 | -1 |  |
| CDS | JPFDDB_23805 | 5160222 | 5161557 | -1 | Metallophosphoesterase |
| gene | JPFDDB_23810 | 5161783 | 5162788 | -1 |  |
| CDS | JPFDDB_23810 | 5161783 | 5162788 | -1 | DUF6634 domain-containing protein |
| gene | JPFDDB_23815 | 5162822 | 5163296 | 1 |  |
| CDS | JPFDDB_23815 | 5162822 | 5163296 | 1 | DNA-binding XRE family transcriptional regulator |
| gene | JPFDDB_23880 | 5175752 | 5178047 | -1 |  |
| CDS | JPFDDB_23880 | 5175752 | 5178047 | -1 | 2-polyprenyl-3-methyl-5-hydroxy-6-metoxy-1,4-benz oquinol methylase |
| gene | JPFDDB_23885 | 5178108 | 5178276 | -1 |  |
| CDS | JPFDDB_23885 | 5178108 | 5178276 | -1 | hypothetical protein |
| gene | JPFDDB_23910 | 5183491 | 5184766 | 1 |  |
| CDS | JPFDDB_23910 | 5183491 | 5184766 | 1 | ABC transmembrane type-1 domain-containing protein |
| gene | JPFDDB_23910 | 5183491 | 5184766 | 1 |  |
| CDS | JPFDDB_23910 | 5183491 | 5184766 | 1 | ABC transmembrane type-1 domain-containing protein |
| gene | JPFDDB_23915 | 5185087 | 5185924 | 1 |  |
| CDS | JPFDDB_23915 | 5185087 | 5185924 | 1 | hypothetical protein |
| gene | JPFDDB_23920 | 5185981 | 5186380 | 1 |  |
| CDS | JPFDDB_23920 | 5185981 | 5186380 | 1 | hypothetical protein |
| gene | JPFDDB_23925 | 5186586 | 5187963 | 1 |  |
| CDS | JPFDDB_23925 | 5186586 | 5187963 | 1 | DUF2397 domain-containing protein |
| gene | JPFDDB_23930 | 5188215 | 5188713 | 1 |  |
| CDS | JPFDDB_23930 | 5188215 | 5188713 | 1 | HTH HARE-type domain-containing protein |
| gene | JPFDDB_23935 | 5189172 | 5189325 | 1 |  |
| CDS | JPFDDB_23935 | 5189172 | 5189325 | 1 | hypothetical protein |
| gene | JPFDDB_23940 | 5189416 | 5190580 | 1 |  |
| CDS | JPFDDB_23940 | 5189416 | 5190580 | 1 | Integrase catalytic domain-containing protein |
| gene | JPFDDB_24915 | 5387279 | 5388548 | 1 |  |
| CDS | JPFDDB_24915 | 5387279 | 5388548 | 1 | Sulfide dehydrogenase [flavocytochrome c] flavoprotein chain |
| gene | JPFDDB_24985 | 5400389 | 5400803 | -1 |  |
| CDS | JPFDDB_24985 | 5400389 | 5400803 | -1 | hypothetical protein |
| gene | JPFDDB_24990 | 5400813 | 5401134 | -1 |  |
| CDS | JPFDDB_24990 | 5400813 | 5401134 | -1 | hypothetical protein |
| gene | JPFDDB_25275 | 5452991 | 5453420 | -1 |  |
| CDS | JPFDDB_25275 | 5452991 | 5453420 | -1 | hypothetical protein |
| gene | JPFDDB_25385 | 5477563 | 5479627 | 1 |  |
| CDS | JPFDDB_25385 | 5477563 | 5479627 | 1 | magnesium chelatase subunit D |
| gene | JPFDDB_25385 | 5477563 | 5479627 | 1 |  |
| CDS | JPFDDB_25385 | 5477563 | 5479627 | 1 | magnesium chelatase subunit D |
| gene | JPFDDB_25400 | 5482095 | 5483175 | 1 |  |
| CDS | JPFDDB_25400 | 5482095 | 5483175 | 1 | All-trans-phytoene synthase/15-cis-phytoene synthase |
| gene | JPFDDB_25405 | 5483197 | 5483620 | -1 |  |
| CDS | JPFDDB_25405 | 5483197 | 5483620 | -1 | PIN domain-containing protein |
| gene | JPFDDB_25430 | 5487751 | 5488906 | 1 |  |
| CDS | JPFDDB_25430 | 5487751 | 5488906 | 1 | Demethylspheroidene O-methyltransferase |
| gene | JPFDDB_25480 | 5496762 | 5497866 | 1 |  |
| CDS | JPFDDB_25480 | 5496762 | 5497866 | 1 | c-type cytochrome |
| gene | JPFDDB_25560 | 5512335 | 5512412 | -1 |  |
| tRNA | JPFDDB_25560 | 5512335 | 5512412 | -1 | tRNA-Ile(gat) |
| gene | JPFDDB_25625 | 5527009 | 5529277 | -1 |  |
| CDS | JPFDDB_25625 | 5527009 | 5529277 | -1 | Ferrichrome-iron receptor |
| gene | JPFDDB_25630 | 5529574 | 5530855 | -1 |  |
| CDS | JPFDDB_25630 | 5529574 | 5530855 | -1 | AB hydrolase-1 domain-containing protein |
| gene | JPFDDB_25635 | 5531111 | 5531363 | 1 |  |
| CDS | JPFDDB_25635 | 5531111 | 5531363 | 1 | hypothetical protein |
| gene | JPFDDB_25640 | 5531491 | 5533279 | 1 |  |
| CDS | JPFDDB_25640 | 5531491 | 5533279 | 1 | outer membrane beta-barrel protein |
| gene | JPFDDB_25785 | 5558176 | 5558947 | -1 |  |
| CDS | JPFDDB_25785 | 5558176 | 5558947 | -1 | HTH HARE-type domain-containing protein |
| gene | JPFDDB_25790 | 5559155 | 5560253 | -1 |  |
| CDS | JPFDDB_25790 | 5559155 | 5560253 | -1 | Plasmid encoded RepA protein |
| gene | JPFDDB_25795 | 5560988 | 5561825 | 1 |  |
| CDS | JPFDDB_25795 | 5560988 | 5561825 | 1 | nucleoside triphosphate pyrophosphohydrolase |
| gene | JPFDDB_25850 | 5570826 | 5572041 | 1 |  |
| CDS | JPFDDB_25850 | 5570826 | 5572041 | 1 | 2-octaprenylphenol hydroxylase |
| gene | JPFDDB_25855 | 5572152 | 5572497 | -1 |  |
| CDS | JPFDDB_25855 | 5572152 | 5572497 | -1 | Response regulatory domain-containing protein |
| gene | JPFDDB_25860 | 5572847 | 5574284 | -1 |  |
| CDS | JPFDDB_25860 | 5572847 | 5574284 | -1 | AMP-binding protein |
| gene | JPFDDB_26090 | 5621504 | 5622434 | 1 |  |
| CDS | JPFDDB_26090 | 5621504 | 5622434 | 1 | Transporter YfdV |
| gene | JPFDDB_26095 | 5622459 | 5622660 | -1 |  |
| CDS | JPFDDB_26095 | 5622459 | 5622660 | -1 | hypothetical protein |
| gene | JPFDDB_26100 | 5622723 | 5623011 | -1 |  |
| CDS | JPFDDB_26100 | 5622723 | 5623011 | -1 | hypothetical protein |
| gene | JPFDDB_26105 | 5623152 | 5627502 | -1 |  |
| CDS | JPFDDB_26105 | 5623152 | 5627502 | -1 | Translocation and assembly module TamB |
| gene | JPFDDB_26170 | 5641053 | 5641737 | -1 |  |
| CDS | JPFDDB_26170 | 5641053 | 5641737 | -1 | PspA/IM30 family protein |
| gene | JPFDDB_26245 | 5656763 | 5658860 | 1 |  |
| CDS | JPFDDB_26245 | 5656763 | 5658860 | 1 | p-hydroxybenzoic acid efflux pump subunit AaeB |
| gene | JPFDDB_26250 | 5658856 | 5659066 | 1 |  |
| CDS | JPFDDB_26250 | 5658856 | 5659066 | 1 | Protein AaeX |
| gene | JPFDDB_26255 | 5659079 | 5660000 | 1 |  |
| CDS | JPFDDB_26255 | 5659079 | 5660000 | 1 | p-hydroxybenzoic acid efflux pump subunit AaeA |
| gene | JPFDDB_26260 | 5660039 | 5661794 | -1 |  |
| CDS | JPFDDB_26260 | 5660039 | 5661794 | -1 | histidine kinase |
| gene | JPFDDB_26310 | 5670224 | 5671271 | 1 |  |
| CDS | JPFDDB_26310 | 5670224 | 5671271 | 1 | putative 2-(5''-triphosphoribosyl)-3'-dephosphocoenzyme-A synthase |
| gene | JPFDDB_26360 | 5682650 | 5684300 | -1 |  |
| CDS | JPFDDB_26360 | 5682650 | 5684300 | -1 | exodeoxyribonuclease VII large subunit |
| gene | JPFDDB_26440 | 5700448 | 5703886 | 1 |  |
| CDS | JPFDDB_26440 | 5700448 | 5703886 | 1 | DNA translocase FtsK |
| gene | JPFDDB_26565 | 5727044 | 5728685 | 1 |  |
| CDS | JPFDDB_26565 | 5727044 | 5728685 | 1 | ATP-dependent RNA helicase RhlE |
| gene | JPFDDB_26725 | 5755326 | 5757063 | -1 |  |
| CDS | JPFDDB_26725 | 5755326 | 5757063 | -1 | flagellar hook-length control protein FliK |
| gene | JPFDDB_26730 | 5757059 | 5758307 | -1 |  |
| CDS | JPFDDB_26730 | 5757059 | 5758307 | -1 | Chemotaxis protein MotC |
| gene | JPFDDB_26740 | 5759699 | 5760554 | -1 |  |
| CDS | JPFDDB_26740 | 5759699 | 5760554 | -1 | Flagellar assembly protein FliH/Type III secretion system HrpE domain-containing protein |
| gene | JPFDDB_26805 | 5769925 | 5772712 | 1 |  |
| CDS | JPFDDB_26805 | 5769925 | 5772712 | 1 | Heparinase II N-terminal domain-containing protein |
| gene | JPFDDB_26850 | 5778780 | 5779362 | -1 |  |
| CDS | JPFDDB_26850 | 5778780 | 5779362 | -1 | flagellar biosynthesis repressor FlbT |
| gene | JPFDDB_26895 | 5787388 | 5787985 | -1 |  |
| CDS | JPFDDB_26895 | 5787388 | 5787985 | -1 | Type II secretion system protein GspC N-terminal domain-containing protein |
| gene | JPFDDB_26935 | 5795127 | 5795988 | 1 |  |
| CDS | JPFDDB_26935 | 5795127 | 5795988 | 1 | Rhamnolipids biosynthesis 3-oxoacyl-[acyl-carrier-protein] reductase |
| gene | JPFDDB_27080 | 5825264 | 5825984 | 1 |  |
| CDS | JPFDDB_27080 | 5825264 | 5825984 | 1 | (5-formylfuran-3-yl)methyl phosphate synthase |
| gene | JPFDDB_27155 | 5840349 | 5841564 | -1 |  |
| CDS | JPFDDB_27155 | 5840349 | 5841564 | -1 | Major facilitator superfamily (MFS) profile domain-containing protein |
| gene | JPFDDB_27210 | 5854809 | 5855319 | 1 |  |
| CDS | JPFDDB_27210 | 5854809 | 5855319 | 1 | TIGR04222 domain-containing protein |
| gene | JPFDDB_27215 | 5855318 | 5856386 | 1 |  |
| CDS | JPFDDB_27215 | 5855318 | 5856386 | 1 | cytochrome c oxidase subunit II |
| gene | JPFDDB_27540 | 5909437 | 5910661 | 1 |  |
| CDS | JPFDDB_27540 | 5909437 | 5910661 | 1 | Tlde1 domain-containing protein |
| gene | JPFDDB_27565 | 5913460 | 5914345 | 1 |  |
| CDS | JPFDDB_27565 | 5913460 | 5914345 | 1 | alpha/beta hydrolase |

**Supplementary Table 2.** The list of protein-coding sequences unique to either resequenced (namely MsGoBio) or original (namely SB0023/3) genome assembly of the *M. symbioticum* SB0023/3 based on the panaroo pangenome analysis.

| **CDS unique to** | **Gene** | **Predicted CDS product** | **Locus tag** | **Contig** | **Contig length (bp)** |
| --- | --- | --- | --- | --- | --- |
| MsGoBio | *group_2421* | chemotaxis protein CheW | JPFDDB_00005 | chromosome | 5964341 |
| MsGoBio | *cheR* | protein-glutamate O-methyltransferase CheR | JPFDDB_00010 | chromosome | 5964341 |
| MsGoBio | *group_2295* | chemotaxis protein CheW | JPFDDB_00015 | chromosome | 5964341 |
| MsGoBio | *group_321* | Frizzy aggregation protein FrzCD | JPFDDB_00020 | chromosome | 5964341 |
| MsGoBio | *group_125* | response regulator | JPFDDB_00025 | chromosome | 5964341 |
| MsGoBio | *group_168* | pseudouridine synthase | JPFDDB_00560 | chromosome | 5964341 |
| MsGoBio | *group_3090* | Integrase catalytic domain-containing protein | JPFDDB_01050 | chromosome | 5964341 |
| MsGoBio | *group_328* | Porin | JPFDDB_01225 | chromosome | 5964341 |
| MsGoBio | *group_3642* | Outer membrane protein beta-barrel domain-containing protein | JPFDDB_01560 | chromosome | 5964341 |
| MsGoBio | *group_422* | Peptidase M15A C-terminal domain-containing protein | JPFDDB_01730 | chromosome | 5964341 |
| MsGoBio | *ftsY* | signal recognition particle-docking protein FtsY | JPFDDB_02045 | chromosome | 5964341 |
| MsGoBio | *group_519* | DUF2336 domain-containing protein | JPFDDB_02060 | chromosome | 5964341 |
| MsGoBio | *group_1090* | Transglycosylase SLT domain-containing protein | JPFDDB_02065 | chromosome | 5964341 |
| MsGoBio | *group_2084* | aldolase/citrate lyase family protein | JPFDDB_02070 | chromosome | 5964341 |
| MsGoBio | *group_1182* | tRNA-dihydrouridine synthase | JPFDDB_02270 | chromosome | 5964341 |
| MsGoBio | *recF* | DNA replication and repair protein RecF | JPFDDB_02590 | chromosome | 5964341 |
| MsGoBio | *ruvB* | Holliday junction branch migration DNA helicase RuvB | JPFDDB_02700 | chromosome | 5964341 |
| MsGoBio | *degP* | Periplasmic serine endoprotease DegP | JPFDDB_02755 | chromosome | 5964341 |
| MsGoBio | *group_20* | Chromosome partition protein Smc | JPFDDB_02765 | chromosome | 5964341 |
| MsGoBio | *group_1466* | Outer membrane protein beta-barrel domain-containing protein | JPFDDB_03370 | chromosome | 5964341 |
| MsGoBio | *ftsI* | Putative peptidoglycan DD-transpeptidase FtsI | JPFDDB_03870 | chromosome | 5964341 |
| MsGoBio | *group_225* | DNA polymerase III subunit gamma/tau | JPFDDB_04690 | chromosome | 5964341 |
| MsGoBio | *group_3643* | IS110 family transposase | JPFDDB_05395 | chromosome | 5964341 |
| MsGoBio | *group_3644* | Glutathione-independent formaldehyde dehydrogenase | JPFDDB_05540 | chromosome | 5964341 |
| MsGoBio | *group_1823* | Alkyl hydroperoxide reductase subunit F | JPFDDB_05815 | chromosome | 5964341 |
| MsGoBio | *group_1614* | hypothetical protein | JPFDDB_06925 | chromosome | 5964341 |
| MsGoBio | *group_782* | CCA-adding enzyme | JPFDDB_07770 | chromosome | 5964341 |
| MsGoBio | *group_3645* | IS110 family transposase | JPFDDB_09025 | chromosome | 5964341 |
| MsGoBio | *pxpB* | 5-oxoprolinase subunit PxpB | JPFDDB_09745 | chromosome | 5964341 |
| MsGoBio | *msuE* | FMN reductase | JPFDDB_10495 | chromosome | 5964341 |
| MsGoBio | *tssA* | type VI secretion system protein TssA | JPFDDB_10870 | chromosome | 5964341 |
| MsGoBio | *group_9* | Heme/hemopexin-binding protein | JPFDDB_10920 | chromosome | 5964341 |
| MsGoBio | *esiB* | Secretory immunoglobulin A-binding protein EsiB | JPFDDB_10925 | chromosome | 5964341 |
| MsGoBio | *nagA* | N-acetylglucosamine-6-phosphate deacetylase | JPFDDB_11060 | chromosome | 5964341 |
| MsGoBio | *group_1211* | Glutamine--fructose-6-phosphate aminotransferase [isomerizing] | JPFDDB_11065 | chromosome | 5964341 |
| MsGoBio | *group_1233* | DUF1311 domain-containing protein | JPFDDB_11320 | chromosome | 5964341 |
| MsGoBio | *terC* | Integral membrane protein TerC | JPFDDB_11360 | chromosome | 5964341 |
| MsGoBio | *group_409* | putative periplasmic serine endoprotease DegP-like | JPFDDB_11975 | chromosome | 5964341 |
| MsGoBio | *lpxK* | tetraacyldisaccharide 4'-kinase | JPFDDB_12375 | chromosome | 5964341 |
| MsGoBio | *cycH* | Cytochrome c-type biogenesis protein CycH | JPFDDB_12760 | chromosome | 5964341 |
| MsGoBio | *group_1273* | hypothetical protein | JPFDDB_13280 | chromosome | 5964341 |
| MsGoBio | *group_55* | Ribonuclease E | JPFDDB_13285 | chromosome | 5964341 |
| MsGoBio | *group_3646* | IS110 family transposase | JPFDDB_14590 | chromosome | 5964341 |
| MsGoBio | *group_648* | UDP-glucose 6-dehydrogenase | JPFDDB_14670 | chromosome | 5964341 |
| MsGoBio | *group_888* | Glycosyltransferase 2-like domain-containing protein | JPFDDB_14820 | chromosome | 5964341 |
| MsGoBio | *group_1033* | molybdopterin-binding protein | JPFDDB_14840 | chromosome | 5964341 |
| MsGoBio | *group_1109* | Holliday junction branch migration DNA helicase RuvB | JPFDDB_15530 | chromosome | 5964341 |
| MsGoBio | *mcrA* | 5-methylcytosine-specific restriction endonuclease McrA | JPFDDB_16265 | chromosome | 5964341 |
| MsGoBio | *tqsA* | AI-2 transport protein TqsA | JPFDDB_16440 | chromosome | 5964341 |
| MsGoBio | *group_2632* | Integrase core domain-containing protein | JPFDDB_16880 | chromosome | 5964341 |
| MsGoBio | *group_3647* | IS256 family ISMex14 transposase | JPFDDB_17185 | chromosome | 5964341 |
| MsGoBio | *group_556* | Ribosomal large subunit pseudouridine synthase C | JPFDDB_17740 | chromosome | 5964341 |
| MsGoBio | *tuf* | elongation factor Tu | JPFDDB_18300 | chromosome | 5964341 |
| MsGoBio | *group_223* | 1-deoxy-D-xylulose-5-phosphate synthase | JPFDDB_19475 | chromosome | 5964341 |
| MsGoBio | *group_889* | polysaccharide biosynthesis/export family protein | JPFDDB_19525 | chromosome | 5964341 |
| MsGoBio | *group_597* | UDP-glucose 6-dehydrogenase | JPFDDB_19540 | chromosome | 5964341 |
| MsGoBio | *group_840* | ABC-2 type transporter transmembrane domain-containing protein | JPFDDB_20330 | chromosome | 5964341 |
| MsGoBio | *group_66* | Aerotolerance regulator N-terminal domain-containing protein | JPFDDB_20430 | chromosome | 5964341 |
| MsGoBio | *dxs* | 1-deoxy-D-xylulose-5-phosphate synthase | JPFDDB_20975 | chromosome | 5964341 |
| MsGoBio | *group_589* | SPOR domain-containing protein | JPFDDB_21185 | chromosome | 5964341 |
| MsGoBio | *group_524* | flagellar hook-length control protein FliK | JPFDDB_21300 | chromosome | 5964341 |
| MsGoBio | *group_446* | DNA polymerase Y family protein | JPFDDB_21460 | chromosome | 5964341 |
| MsGoBio | *imuA* | ImuA protein | JPFDDB_21465 | chromosome | 5964341 |
| MsGoBio | *group_1600* | collagen-like protein | JPFDDB_21470 | chromosome | 5964341 |
| MsGoBio | *clcA* | H(+)/Cl(-) exchange transporter ClcA | JPFDDB_21475 | chromosome | 5964341 |
| MsGoBio | *group_2278* | Beta-Ig-H3/fasciclin | JPFDDB_21480 | chromosome | 5964341 |
| MsGoBio | *group_1867* | anti-sigma factor | JPFDDB_21485 | chromosome | 5964341 |
| MsGoBio | *group_893* | polysaccharide biosynthesis/export family protein | JPFDDB_21580 | chromosome | 5964341 |
| MsGoBio | *group_21* | Tetratricopeptide repeat protein | JPFDDB_22195 | chromosome | 5964341 |
| MsGoBio | *group_844* | BioF2-like acetyltransferase domain-containing protein | JPFDDB_22200 | chromosome | 5964341 |
| MsGoBio | *group_122* | Polysaccharide chain length determinant N-terminal domain-containing protein | JPFDDB_22205 | chromosome | 5964341 |
| MsGoBio | *group_3648* | IS110 family transposase | JPFDDB_22530 | chromosome | 5964341 |
| MsGoBio | *group_3649* | nitrate ABC transporter permease | JPFDDB_22675 | chromosome | 5964341 |
| MsGoBio | *group_115* | DUF5682 domain-containing protein | JPFDDB_22875 | chromosome | 5964341 |
| MsGoBio | *group_762* | lytic murein transglycosylase | JPFDDB_22935 | chromosome | 5964341 |
| MsGoBio | *group_3650* | IS256 family ISMex14 transposase | JPFDDB_23195 | chromosome | 5964341 |
| MsGoBio | *group_2509* | Secreted protein | JPFDDB_23305 | chromosome | 5964341 |
| MsGoBio | *group_2634* | Secreted protein | JPFDDB_23405 | chromosome | 5964341 |
| MsGoBio | *group_185* | DNA repair exonuclease SbcCD ATPase subunit | JPFDDB_23465 | chromosome | 5964341 |
| MsGoBio | *group_3651* | IS110 family transposase | JPFDDB_23745 | chromosome | 5964341 |
| MsGoBio | *group_606* | Metallophosphoesterase | JPFDDB_23805 | chromosome | 5964341 |
| MsGoBio | *group_1197* | DUF6634 domain-containing protein | JPFDDB_23810 | chromosome | 5964341 |
| MsGoBio | *group_2486* | DNA-binding XRE family transcriptional regulator | JPFDDB_23815 | chromosome | 5964341 |
| MsGoBio | *ubiG* | 2-polyprenyl-3-methyl-5-hydroxy-6-metoxy-14-benzoquinol methylase | JPFDDB_23880 | chromosome | 5964341 |
| MsGoBio | *group_3525* | hypothetical protein | JPFDDB_23885 | chromosome | 5964341 |
| MsGoBio | *group_703* | ABC transmembrane type-1 domain-containing protein | JPFDDB_23910 | chromosome | 5964341 |
| MsGoBio | *group_1606* | hypothetical protein | JPFDDB_23915 | chromosome | 5964341 |
| MsGoBio | *group_2729* | hypothetical protein | JPFDDB_23920 | chromosome | 5964341 |
| MsGoBio | *group_557* | DUF2397 domain-containing protein | JPFDDB_23925 | chromosome | 5964341 |
| MsGoBio | *group_2411* | HTH HARE-type domain-containing protein | JPFDDB_23930 | chromosome | 5964341 |
| MsGoBio | *group_3552* | hypothetical protein | JPFDDB_23935 | chromosome | 5964341 |
| MsGoBio | *group_901* | Integrase catalytic domain-containing protein | JPFDDB_23940 | chromosome | 5964341 |
| MsGoBio | *group_331* | Porin | JPFDDB_24625 | chromosome | 5964341 |
| MsGoBio | *group_598* | UDP-glucose 6-dehydrogenase | JPFDDB_24860 | chromosome | 5964341 |
| MsGoBio | *group_2683* | hypothetical protein | JPFDDB_24985 | chromosome | 5964341 |
| MsGoBio | *group_187* | magnesium chelatase subunit D | JPFDDB_25385 | chromosome | 5964341 |
| MsGoBio | *group_2650* | PIN domain-containing protein | JPFDDB_25405 | chromosome | 5964341 |
| MsGoBio | *group_915* | Demethylspheroidene O-methyltransferase | JPFDDB_25430 | chromosome | 5964341 |
| MsGoBio | *group_1006* | c-type cytochrome | JPFDDB_25480 | chromosome | 5964341 |
| MsGoBio | *group_695* | AB hydrolase-1 domain-containing protein | JPFDDB_25630 | chromosome | 5964341 |
| MsGoBio | *group_3223* | hypothetical protein | JPFDDB_25635 | chromosome | 5964341 |
| MsGoBio | *group_1748* | HTH HARE-type domain-containing protein | JPFDDB_25785 | chromosome | 5964341 |
| MsGoBio | *repA* | Plasmid encoded RepA protein | JPFDDB_25790 | chromosome | 5964341 |
| MsGoBio | *mazG* | nucleoside triphosphate pyrophosphohydrolase | JPFDDB_25795 | chromosome | 5964341 |
| MsGoBio | *group_798* | 2-octaprenylphenol hydroxylase | JPFDDB_25850 | chromosome | 5964341 |
| MsGoBio | *group_2891* | Response regulatory domain-containing protein | JPFDDB_25855 | chromosome | 5964341 |
| MsGoBio | *group_489* | AMP-binding protein | JPFDDB_25860 | chromosome | 5964341 |
| MsGoBio | *group_421* | Glucans biosynthesis protein G | JPFDDB_25970 | chromosome | 5964341 |
| MsGoBio | *group_667* | Adenylate cyclase 1 | JPFDDB_25975 | chromosome | 5964341 |
| MsGoBio | *group_1354* | Transporter YfdV | JPFDDB_26090 | chromosome | 5964341 |
| MsGoBio | *group_3432* | hypothetical protein | JPFDDB_26095 | chromosome | 5964341 |
| MsGoBio | *group_3078* | hypothetical protein | JPFDDB_26100 | chromosome | 5964341 |
| MsGoBio | *aaeX* | Protein AaeX | JPFDDB_26250 | chromosome | 5964341 |
| MsGoBio | *group_1374* | p-hydroxybenzoic acid efflux pump subunit AaeA | JPFDDB_26255 | chromosome | 5964341 |
| MsGoBio | *xseA* | exodeoxyribonuclease VII large subunit | JPFDDB_26360 | chromosome | 5964341 |
| MsGoBio | *fliK* | flagellar hook-length control protein FliK | JPFDDB_26725 | chromosome | 5964341 |
| MsGoBio | *gspC* | Type II secretion system protein GspC N-terminal domain-containing protein | JPFDDB_26895 | chromosome | 5964341 |
| MsGoBio | *group_1911* | (5-formylfuran-3-yl)methyl phosphate synthase | JPFDDB_27080 | chromosome | 5964341 |
| MsGoBio | *group_3652* | Abasic site processing protein | JPFDDB_27425 | chromosome | 5964341 |
| MsGoBio | *yedK* | SOS response-associated peptidase YedK | JPFDDB_27430 | chromosome | 5964341 |
| MsGoBio | *group_784* | Tlde1 domain-containing protein | JPFDDB_27540 | chromosome | 5964341 |
| MsGoBio | *group_3490* | hypothetical protein | JPFDDB_27720 | chromosome | 5964341 |
| MsGoBio | *group_3653* | Abasic site processing protein | JPFDDB_27745 | chromosome | 5964341 |
| MsGoBio | *group_3620* | hypothetical protein | JPFDDB_27750 | chromosome | 5964341 |
| MsGoBio | *group_3654* | SOS response-associated peptidase YedK | JPFDDB_27755 | chromosome | 5964341 |
| MsGoBio | *group_1815* | Integrase catalytic domain-containing protein | JPFDDB_27760 | chromosome | 5964341 |
| MsGoBio | *group_4143* | hypothetical protein | JPFDDB_27850 | pMSB0023-3_1 | 123595 |
| MsGoBio | *group_4160* | IS256 family transposase | JPFDDB_27855 | pMSB0023-3_1 | 123595 |
| MsGoBio | *group_4136* | HTH-like domain-containing protein | JPFDDB_27965 | pMSB0023-3_1 | 123595 |
| MsGoBio | *group_4161* | Transposase IS3/IS911 family protein | JPFDDB_27970 | pMSB0023-3_1 | 123595 |
| MsGoBio | *group_4162* | IS3 family transposase | JPFDDB_27975 | pMSB0023-3_1 | 123595 |
| MsGoBio | *group_4163* | ParA family protein | JPFDDB_28010 | pMSB0023-3_1 | 123595 |
| MsGoBio | *group_4188* | Translation initiation factor IF-2 | JPFDDB_28015 | pMSB0023-3_1 | 123595 |
| MsGoBio | *group_4164* | IS3 family transposase | JPFDDB_28080 | pMSB0023-3_1 | 123595 |
| MsGoBio | *group_4165* | Transposase IS3/IS911 family protein | JPFDDB_28085 | pMSB0023-3_1 | 123595 |
| MsGoBio | *group_4226* | IS256 family ISMex14 transposase | JPFDDB_28500 | pMSB0023-3_2 | 57877 |
| MsGoBio | *group_4271* | hypothetical protein | JPFDDB_28505 | pMSB0023-3_2 | 57877 |
| MsGoBio | *group_4249* | Integrase catalytic domain-containing protein | JPFDDB_28510 | pMSB0023-3_2 | 57877 |
| MsGoBio | *group_4238* | Winged helix-turn helix domain-containing protein | JPFDDB_28515 | pMSB0023-3_2 | 57877 |
| MsGoBio | *group_4260* | IS110 family transposase | JPFDDB_28520 | pMSB0023-3_2 | 57877 |
| MsGoBio | *group_4227* | IS110 family transposase | JPFDDB_28525 | pMSB0023-3_2 | 57877 |
| MsGoBio | *group_4223* | Helix-turn-helix domain-containing protein | JPFDDB_28600 | pMSB0023-3_2 | 57877 |
| MsGoBio | *group_4228* | IS110 family transposase | JPFDDB_28605 | pMSB0023-3_2 | 57877 |
| MsGoBio | *group_4242* | hypothetical protein | JPFDDB_28675 | pMSB0023-3_2 | 57877 |
| SB0023/3 | *group_4358* | hypothetical protein | MET9862_RS29065 | NZ_CABFPH010000244 | 775 |
| SB0023/3 | *group_4357* | methyl-accepting chemotaxis protein | MET9862_RS28935 | NZ_CABFPH010000222 | 1901 |
| SB0023/3 | *group_4356* | hypothetical protein | MET9862_RS28905 | NZ_CABFPH010000219 | 2003 |
| SB0023/3 | *group_4355* | MBG domain-containing protein | MET9862_RS28370 | NZ_CABFPH010000190 | 4908 |
| SB0023/3 | *group_4354* | caspase family protein | MET9862_RS28365 | NZ_CABFPH010000190 | 4908 |
| SB0023/3 | *aadA1* | ANT(3'')-Ia family aminoglycoside nucleotidyltransferase AadA1 | MET9862_RS28185 | NZ_CABFPH010000183 | 5573 |
| SB0023/3 | *group_3209* | hypothetical protein | MET9862_RS16030 | NZ_CABFPH010000046 | 42168 |
